# Polycomb Protein Eed is Required for Neurogenesis and Cortical Injury Activation in the Subventricular Zone

**DOI:** 10.1093/cercor/bhx289

**Published:** 2018-02-03

**Authors:** Bin Sun, Eunhyuk Chang, Anna Gerhartl, Francis G Szele

**Affiliations:** Department of Physiology, Anatomy and Genetics, University of Oxford, Oxford, UK

**Keywords:** adult neurogenesis, epigenetics, PRC2, subventricular zone

## Abstract

The postnatal subventricular zone (SVZ) harbors neural stem cells (NSCs) that exhibit robust neurogenesis. However, the epigenetic mechanisms that maintain NSCs and regulate neurogenesis remain unclear. We report that label-retaining SVZ NSCs express Eed, the core component of Polycomb repressive complex 2. In vivo and in vitro conditional knockout and knockdown show Eed is necessary for maintaining NSC proliferation, neurogenesis and neurosphere formation. We discovered that Eed functions to maintain p21 protein levels in NSCs by repressing Gata6 transcription. Both Gata6 overexpression and p21 knockdown reduced neurogenesis, while Gata6 knockdown or p21 overexpression partially rescued neurogenesis after Eed loss. Furthermore, genetic deletion of Eed impaired injury induced SVZ proliferation and emigration. These data reveal a novel epigenetic regulated pathway and suggest an essential role for Eed in SVZ homeostasis and injury.

## Introduction

The postnatal/adult subventricular zone (SVZ) harbors the largest population of neural stem cells (NSCs) in the brain (Ihrie and Alvarez-Buylla 2011). SVZ NSCs self-renew slowly throughout life and generate rapidly dividing transit amplifying progenitors (TAPs), which give rise to neuroblasts that migrate to the olfactory bulbs (OB) in the rostral migratory stream (RMS). SVZ lineage progresses along a gradual continuum (Llorens-Bobadilla et al. 2015) and cells can be distinguished by morphology, position, marker expression, and behavior (Doetsch et al. 1999; Mamber et al. 2013). The SVZ is an excellent system for uncovering developmental mechanisms and has therapeutic relevance, since progenitors migrate to injuries and provide neuroprotection (Pluchino et al. 2003; Young et al. 2011). SVZ NSCs can also become harmful when disruption of tightly regulated SVZ cell cycles leads to gliomagenesis (Sanai et al. 2005; Bardella et al. 2016). Therefore, understanding what mechanisms regulate activation of stem cell division and neurogenesis in SVZ cells is a fundamental developmental question which could yield therapeutic approaches.

Multiple cell cycle activators and inhibitors sustain SVZ NSC quiescence and facilitate a “ready for activation” state (Cheung and Rando 2013). The cyclin-dependent kinase inhibitor (CDKI) Cdkn1a encodes p21, which counteracts the cyclin-dependent kinases, Cdk1 and Cdk2. SVZ NSCs express p21, which is required for their quiescence (Kippin et al. 2005; Porlan et al. 2013). Cdkn2a encodes the CDKIs p16Ink4a and p19Arf (Kamijo et al. 1997), which also limit SVZ NSC self-renewal (Nishino et al. 2008). Epigenetic mechanisms regulate embryonic and adult NSC cell cycle length, stem cell activation and lineage progression via CDKIs. The epigenetic Polycomb repressive complex 1 (PRC1) component Bmi1 represses p16Ink4a, p19Arf and p21, to promote proliferation in the adult SVZ (Molofsky et al. 2003; Fasano et al. 2007). Since most SVZ NSCs are quiescent in vivo, these studies may have characterized PRC1 functions in TAPs rather than in NSCs. Yet, p21 is expressed in SVZ NSCs in vivo and is important for NSC quiescence and maintenance (Porlan et al. 2013). The question of how CDKI expression is balanced in different cells of the SVZ lineage is important but not yet resolved.

PRC2-mediated gene silencing controls developmental events and adult tissue maintenance through histone methylation (H3K27me3). PRC2 consists of the core protein Eed (Embryonic ectoderm development), and other subunits including the methyltransferase Ezh2 (Enhancer of zeste homolog 2), Suz12 (Suppressor of zeste 12 Homolog), and RbAp46/48 (Retinoblastoma protein associated protein 46/48) (Margueron and Reinberg 2011). PRC2 represses cell-type specific genes during development, and loss of Ezh2 in early embryogenesis disrupts the temporal order of cerebral cortex neuron generation and is necessary for adult neurogenesis (Pereira et al. 2010; Hwang et al. 2014). Although some biochemical features of Eed have been elucidated, its function in neural development and adult stem cells is unidentified. This is important, however, because Eed is an indispensable PRC2 subunit—when it is eliminated, PRC2 can no longer function. In contrast, the Ezh2 homolog Ezh1 can compensate for Ezh2 loss and removing Eed expression eliminates this confounding factor (Shen et al. 2008; Xie et al. 2013).

We first show that Eed is expressed by NSC, upstream in the SVZ lineage compared with Ezh2. We tested Eed function with conditional knockouts, knockdown and overexpression in vivo and in vitro and show it is necessary for SVZ NSC quiescence, maintenance, and neurogenesis. Unlike Ezh2, Eed suppresses Gata6, a transcription factor. Eed loss and increased Gata6 decreases p21 protein levels. These findings suggest that Eed regulation of Gata6 fine-tunes p21 levels and that this is essential for NSC activation and neurogenesis.

## Materials and Methods

### Mice

Glast-CreERT2 (Mori et al. 2006), Eed^fl/fl^ mice (Xie et al. 2013), and Ezh2^fl/fl^ (Su et al. 2003) were maintained on a C57BL/6 background. For Cre induction in postnatal pups, 2 protocols were used: either 0.2 mg/g of tamoxifen (Sigma) was injected subcutaneously at P1 or pups were injected with 0.2 mg/g of tamoxifen at P1 followed with daily injections in the lactating mothers (150 mg/kg) for another 2 days. For Cre induction in adult mice, 150 mg/kg/d tamoxifen was injected intraperitoneally for 5 days. Littermates were used as controls. To label fast proliferating cells, BrdU (Sigma, 50 mg/kg) was injected intraperitoneally 2 h before euthanasia. For label-retaining cells, BrdU (50 mg/kg) was injected every 12 h for 5 days and mice were euthanised 6 weeks after the last injection. All animal procedures were carried out with Oxford University Research Ethics Committee approval in accordance with the Animals (Scientific Procedures) Act of 1986 (UK).

### Postnatal Electroporation

Electroporation was performed as published (Boutin et al. 2008; Chesler et al. 2008). Constructs for electroporation were prepared with Endofree Plasmid Maxi Kit (Qiagen). 0.1% Fast Green was added to plasmids for tracing ventricular injection. P0–P2 pups were anesthetized by hypothermia. A 1–2 μl plasmid solution was injected into the lateral ventricle via a pulled glass capillary. Pups were then subjected to electrical pulses (100 V, 50 ms ON with 850 ms intervals for 5 cycles) by tweezer electrodes coated with conductive gel. The dorsolateral and striatal SVZ was primarily targeted. After surgery, pups were kept at 37 °C in a warming box for recovery and then returned to the dam. Brains were collected at the indicated time points.

### Neurosphere Culture

SVZ tissues were dissected and dissociated with Accutase (Sigma) at 37 °C. Cells were then cultured in Neurobasal medium (Gibco) containing B27 (Gibco), N2 (Invitrogen), l-glutamine (Invitrogen), Pen Strep (Invitrogen), EGF (20 ng/mL, Sigma), and FGF-2 (20 ng/mL, R&D). For bulk culture, cells were plated at 1 × 10^5^/mL in 6-well plates (non-TC, BD). For clonal density culture, cells were seeded at 10/μL in 96-well plates (non-TC) and spheres were counted at 7–8 days. For differentiation, 1 × 10^5^ cells were seeded per well without growth factors in 8-well chamber slides (Nunc) coated with poly-d-lysine (Sigma) and laminin (Invitrogen). Cells were fixed for immunocytochemistry 7 days later.

### ChIP

ChIP was performed as described (Shen et al. 2008, 2009). Rabbit antibodies against H3K27me3 (Millipore 07-449) and mouse antibodies against Eed (Millipore 05-1320) were used for immunoprecipitation. DNA was purified with PCR purification kit (Qiagen) and subjected for quantitative PCR with SYBR Green. Relative enrichment was normalized to the input and the genomic background with the intron of actin as the internal control. The primer sequences are listed in Supplemental Experimental Procedures.

### RT-PCR

Total RNA was obtained from samples with RNeasy Mini Kit (Qiagen) following the manufacturer’s instructions. Genomic DNA was removed with on-column DNase set (Qiagen), and reverse transcription was performed by SuperScript III RT kit (Invitrogen). The cDNA was subjected to real-time quantitative PCR with SYBR Green PCR Master Mix (Kapa Biosystems or Invitrogen). The target mRNAs were normalized relative to 18S ribosomal RNA. The primer sequences are listed in Supplemental Experimental Procedures.

### Cerebral Cortex Lesions

Cortical injuries were generated as described (Goings et al. 2002, 2004). Briefly, 2–3-month-old mice were anesthetized with Ketamine/Xylazine. The left frontoparietal cerebral cortex from Bregma to 1.2 mm anterior to Bregma was aspirated with a fire-polished glass Pasteur pipette connected to a mild vacuum. Mice were monitored postsurgery and euthanised at the indicated time.

### Constructs and Lentivirus Production

pCAG-Cre-IRES2-EGFP (26646), pCAG-IRES2-EGFP (pCAGIG, 11159), pLKO.1-puro (8453), pcDNA1-Gata6 (51929), and p21 shRNA (25868) were from Addgene (Liang et al. 2001; Stewart et al. 2003; Matsuda and Cepko 2004; Woodhead et al. 2006; Fasano et al. 2007). Eed shRNA construct was a kind gift from Prof. Neil Brockdorff (Cooper and Brockdorff 2013). siRNAs against firefly luciferase or Gata6 were purchased from Sigma. pLKO.1-based lentivirus was packaged as previously published (Tavares et al. 2012). Briefly, HEK293FT cells were transfected with pLKO.1, psPAX2 and pMD2.G constructs and fed with fresh medium. Supernatants were collected 48 and 72 h later and centrifuged at 25 000 rpm, 4 °C for 100 min to concentrate the virus.

### Western Blot

Proteins were run in 10% SDS-PAGE gel at 100 V and transferred into PVDF membrane at 100 V for 90 min. The membrane was blocked in 5% skim milk in PBST (PBS+0.1% Tween-20) and incubated with primary antibody at 4 °C overnight. On the second day, the membrane was washed in PBST and incubated with the secondary antibody for 1h. The membrane was washed again in PBST, and chemofluorescence was developed with Lumigen PS-3 kit. All the western blots have been repeated at least 3 times and representative images are shown in the figures.

### Immunohistochemistry, Image Acquisition and Quantification

Thirty micrometer--thick free-floating sections were incubated with indicated primary antibodies overnight at 4 °C. Alexa conjugated secondary antibodies (Invitrogen) were then used for immunofluorescence detection. For BrdU, Ezh2, and Eed immunostaining, sections were treated with 2 M HCL for 1 h at 37 °C for antigen retrieval. Sections were then washed with PBS and processed for blocking. DAPI (Sigma) was used for nuclei staining before mounting. Primary antibodies used are listed in the Supplemental Experimental Procedures. Z-stack images were taken with confocal microscopy (Zeiss 710) to confirm co-localization. At least 4 layers with 2 μm intervals between images were acquired for analysis. The images were processed with Image J. For olfactory bulb quantification, evenly spaced 10 sections per animal were analyzed to cover anterior to posterior OB. For percentage cell quantifications, at least 100 cells were counted but frequently several hundred were quantified.

## Results

### Eed is Expressed in SVZ Stem and Progenitor Cells

**Figure 1.**
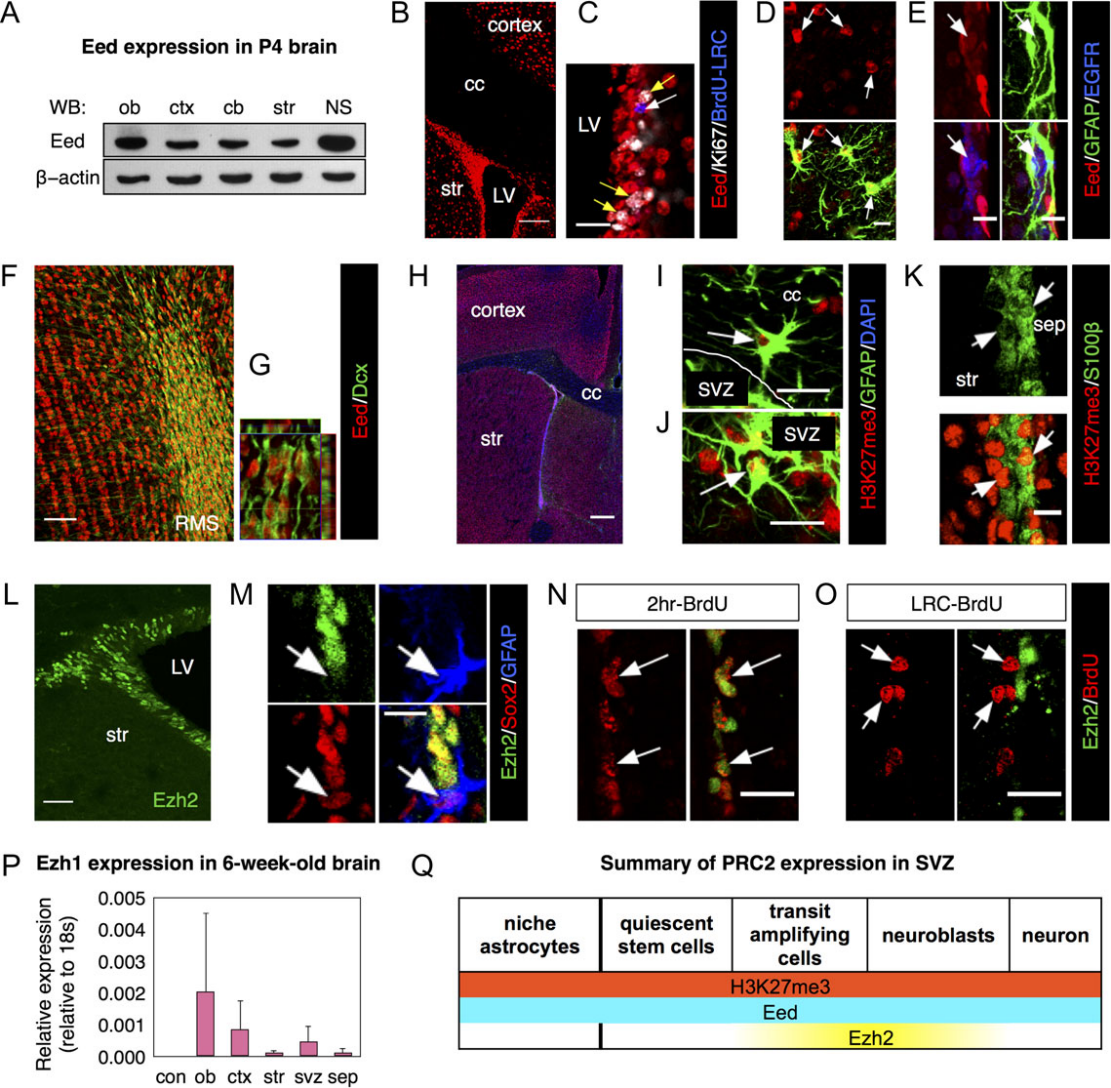
Eed, but not Ezh2, is expressed in the entire SVZ lineage. (*A*) Western blot for Eed in different regions of postnatal brains. Neurospheres (NS) were cultured from P4 SVZ tissues. (*B*, *C*) Expression of Eed in proliferating cells (white arrows) and BrdU+ label-retaining cells (yellow arrows). (*D*–*G*) Eed expression in GFAP+/EGFR− SVZ niche astrocytes (arrows in *D*), TAPs (arrows in *E*) and neuroblasts (*F*–*G*). (*H*–*K*) Immunostaining for H3K27me3 in astrocytes in corpus callosum (arrow in *I*), in SVZ (arrow in *J*) and ependymal cells (arrows in *K*). (*L*) Immunostaining of Ezh2 in the SVZ of the young adult mouse forebrain (6 weeks old). (*M*) Confocal images of Ezh2 in GFAP+/Sox2+ neural stem cells. Arrows in (*M*) indicate GFAP+/Sox2+ stem cells. (*N*, *O*) Images of Ezh2 expression in acutely proliferating SVZ cells (*N*) or label-retaining cells (*O*). Arrows indicate BrdU+ cells. (*P*) qPCR for Ezh1 mRNA expression in different regions of adult brain. *N* = 3. (*Q*) Schematic representation of PRC2 expression in the SVZ neural stem cell lineage. Abbreviations: LV, lateral ventricle; str, striatum; ob, olfactory bulb; ctx, cortex; cb, cerebellum; NS, neurosphere; cc, corpus callosum; sep, septum; RMS, rostral migratory stream. Scale bars represent 10 μm in (*M*), (*D*, *E*), (*K*); 20 μm in (*N*, *O*), (C), (*I*, *J*); 40 μm in (*L*); 50 μm in (*K*); 100 μm in (*B*); 300 μm in (*H*).

We first examined PRC2 component expression patterns. Western blots showed core PRC2 protein, Eed expression in several brain areas at P4 as well as in SVZ-derived neurospheres (Fig. 1*A*). In 6-week-old brains, Eed immunofluorescence was detected in the SVZ, the striatum and cerebral cortex (Fig. 1*B*). All DAPI+ nuclei examined in the SVZ expressed Eed, indicating it is expressed in all cells of the lineage. Importantly, both BrdU+ label-retaining cells (LRC) and Ki67+ actively proliferating cells expressed Eed in the SVZ (Figs 1*C* and S1A and S1B). The former result suggested quiescent NSCs express Eed. EGFR+/GFAP+NSC, EGFR+/GFAP− TAPs and EGFR−/GFAP+ niche astrocytes were all Eed+ (Fig. 1*D*, *E*). In the RMS and OB, Dcx+ neuroblasts and granular layer cells expressed Eed (Fig. 1*F*, *G*). Similar to Eed, PRC2 epigenetic mark H3K27me3+ nuclei were found throughout the brain (Fig. 1*H*). GFAP+ astrocytes in the corpus callosum and SVZ (Fig. 1*I*, *J*), as well as S100β+ niche astrocytes and ependymal cells (Fig. 1*K*) and Dcx+ neuroblasts (Fig. S1C) were all H3K27me3+.

Unlike Eed, Ezh2 immunoreactivity was restricted to the SVZ (Fig. 1*L*) and RMS with few Ezh2+ cells elsewhere. However, only 6.45 ± 2.75% of GFAP+/Sox2+SVZ NSCs were Ezh2+ (Fig. 1*M*). Similarly, only 7.58 ± 1.96% of SVZ LRC had Ezh2+ nuclei (Fig. 1*O*). The majority (97.37 ± 1.96%) of actively proliferating BrdU+ cells were Ezh2+ (Fig. 1*N*), which was confirmed with phospho-histone 3 (PHi3) and Ezh2 costaining (Fig. S1D). Ezh2+ nuclei were mainly detected in doublecortin+ (Dcx) neuroblasts in the RMS and olfactory bulb (OB) (Fig. S1D). Our data showing Dcx+ neuroblasts express Ezh2 is consistent with the literature (Hwang et al. 2014). These data strongly suggest that Ezh2 is expressed in TAPs and neuroblasts but not in SVZ NSCs. The Allen Brain Atlas shows Ezh1 is expressed in cells or regions lacking Ezh2, which would explain the broad distribution of H3K27me3. Ezh1 mRNA was detected in multiple brain regions including the SVZ (Fig. 1*P*). Unfortunately there are no reliable Ezh1 antibodies for immuohistochemistry. However, the transcriptome of FACSorted SVZ cells shows an enrichment of Ezh1 in quiescent SVZ stem cells (type B cells) (Codega et al. 2014). This is consistent with the general idea that Ezh1 is expressed by quiescent stem cells while Ezh2 is expressed by active stem cells (Cheung and Rando 2013). Overall, our data suggests a model in which Eed is expressed in quiescent NSCs as well as after they become activated and enter the neurogenic program (Fig. 1*Q*). In contrast our data suggest Ezh2 is not expressed in SVZ NSC.

### Eed is Required for SVZ Neural Stem Cell Activation

**Figure 2.**
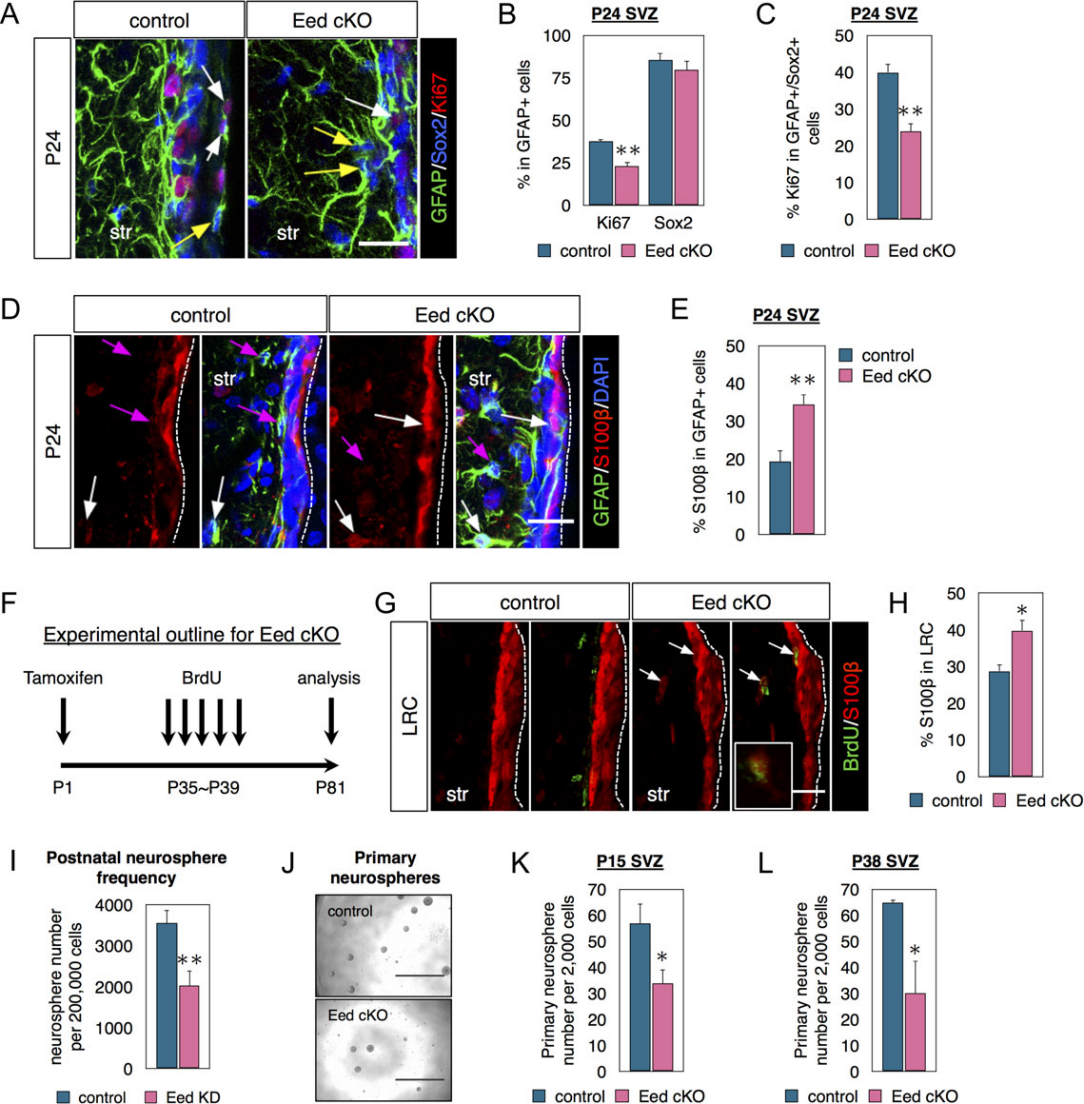
Eed is necessary for NSC activation, maintenance and neurosphere formation. (*A*–*C*) Costaining and quantifications of GFAP, Sox2 and Ki67 in the P24 SVZ of littermate controls and Eed cKO mice. The white arrows indicate Ki67+/Sox2+/GFAP+ cells and the yellow arrows indicate Ki67−/Sox2+/GFAP+ cells. *N* = 3. (*A*–*E*) Tamoxifen given at P1 and brains analyzed at P24. (*D*, *E*) Coimmunostaining and quantifications of GFAP and S100β in the P24 SVZ. The white arrows indicate GFAP+/S100β+ cells, and the magenta arrows indicate GFAP+/S100β- cells. *N* = 3. (*F*) Schematic diagram of the cKO experimental design used in *G*, *H*. (*G*,* H*) Coimmunostaining and quantifications of BrdU and S100β in SVZ from mice injected with BrdU for label-retaining analysis. Arrows indicate BrdU+/S100β+ cells. *N* = 3. (*I*) In vitro knockdown of Eed by lentivirus infection and neurosphere number quantified after 3 days in vitro (DIV). *N* = 3. (*J*, *K*) Primary neurospheres cultured from control or Eed cKO P15 SVZ and counted after 7 DIV. *N* = 5–6. (*L*) Primary neurospheres cultured from control or Eed cKO P38 SVZ and counted after 7 DIV. *N* = 3. Data are shown as mean ± SEM and analyzed by two-tailed Student *t*-test. *P < 0.05, **P < 0.01. Scale bars represent 20 μm in (*A*, *D*, *G*).

We next examined Eed function in the SVZ. We crossed Glast-CreERT2 mice with Eed^fl/fl^ mice (Mori et al. 2006; Xie et al. 2013) to remove Eed function in postnatal SVZ NSCs via tamoxifen (TMX) administration in P0~P1 pups (Materials and Methods). To boost Cre-recombination efficiency, we gave TMX to pups and to lactating dams. Genotyping and RT-PCR confirmed Eed cKO (Fig. S1E, F). At P14, the number of PHi3+ cells was unchanged in Eed cKO SVZs (Fig. S1G). However, by P24 fewer GFAP+ cells were proliferating (Ki67+) (SVZ NSCs and possibly niche astrocytes) in Eed cKOs (Fig. 2*A*, *B*). Further analysis of Eed loss revealed 45% reduction in GFAP+/Sox2+NSCs that expressed Ki67 (Fig. 2*A*, *C*), suggesting fewer stem cells are entering the cell cycle. Ezh2 cKO SVZs did not exhibit such a decrease in GFAP+/Sox2+NSCs that expressed Ki67 (Fig. S2G), supporting the observation that NSCs do not express Ezh2.

Stem cell activation defects or loss of stemness and acquisition of niche astrocyte phenotype could decrease NSC proliferation. The latter was assessed by coimmunostaining GFAP+ cells with S100β, a marker of niche astrocytes (and ependymal cells) (Raponi et al. 2007). Seventy-five percent more GFAP+ cells expressed S100β in the SVZ after Eed loss (Fig. 2*D*, *E*), suggesting loss of stemness and NSC differentiation into niche astrocytes. We next sought to determine if a longer survival time of P81 after Eed cKO would have similar results. For label-retaining analysis, BrdU was given from P35 to P39, and brains collected 6 weeks later (Fig. 2*F*). A smaller percentage of BrdU LRC cells in Eed cKO SVZ expressed immunodetectable H3K27me3, confirming loss of PRC2 function and presence of knockout cells (Fig. S1H, I). Although Eed loss did not significantly affect overall LRC cell numbers (Fig. S1J), more LRCs expressed S100β in Eed cKO mice. This suggests stem cells are less active and the data are consistent with them acquiring a niche astrocyte phenotype (Fig. 2*G*, *H*). Importantly, most S100β+/GFAP+ exhibit long basal processes, and these cells as well as S100β+/LRC cells are primarily within the SVZ body rather than lining the ventricle (Fig. 2*D*,* G*) indicating they are not ependymal cells. Examination of ependymal cells lining the ventricles shows that Eed cKO does not change S100β expression (Fig. 2*G*).

We next examined primary SVZ neurospheres, which can be formed by SVZ NSCs and TAPs. Lentiviral Eed knockdown reduced the number of neurospheres formed 4 days in vitro (div) (Fig. 2*I*). Both P15 and P38 Eed cKO SVZs yielded 50% fewer clonal neurospheres (Fig. 2*J*, *K*, *L*). Together, these data show Eed is required for NSC activation.

### Eed is Required for Postnatal OB Neurogenesis

**Figure 3.**
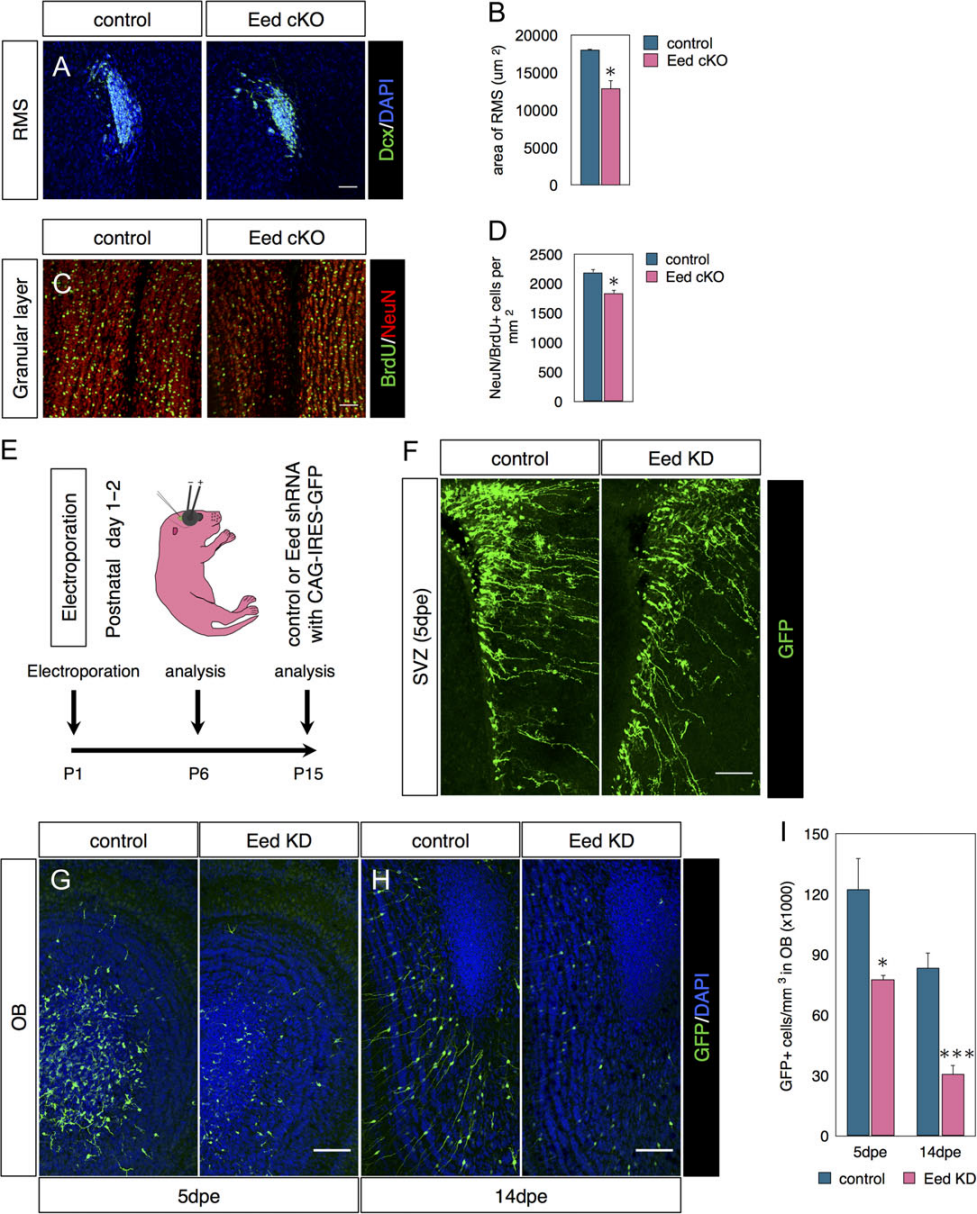
Eed is required for postnatal OB neurogenesis. (*A*–*D*) Eed cKO schedule as in Fig. 2*F*. (*A*) Immunostaining for Dcx in the RMS. (*B*) Quantification of surface area occupied by Dcx+ cells in the RMS. *N* = 3–4. (*C*) Costaining for BrdU and NeuN in the granular layer of the olfactory bulb. (*D*) Quantifications of NeuN+/BrdU+ double labeled cells in the OB. *N* = 3–4. (*E*) Diagram of postnatal electroporation schedule used in *F*–*I*. (*F*) GFP immunofluorescence in SVZ 5 days postelectroporation. (*G*–*J*) Confocal images and quantifications of GFP+ cells in the OB 5 days (*G*) or 14 days (*H*) postelectroporation. *N* = 5-6. Data are shown as mean ± SEM and analyzed by two-tailed Student *t*-test. *P < 0.05, **P < 0.01, ***P < 0.001. Scale bars represent 50 μm in (*A*, *C*), 100 μm (*F*–*H*).

Next, we examined OB neurogenesis in P81 Glast-CreERT;Eed^fl/fl^ mice given TMX as neonates and BrdU between P35-39 (as in Fig. 2*F*). Eed cKOs had reduced RMS surface areas (Fig. 3*A*, *B*) and fewer NeuN+/BrdU+ newborn neurons in the OB compared with controls (Fig. 3*C*, *D*). Similar experiments in Glast-CreERT; Ezh2^fl/fl^ mice also resulted in decreased neurogenesis (Fig. S2 and Supplementary Results), comparable to a previous study (Hwang et al. 2014).

Glast-CreERT2-mediated recombination occurs in SVZ NSCs as well as in astrocytes outside the SVZ (Mori et al. 2006). Thus it was possible that Eed cKO in non-SVZ astrocytes affected the number of OB NeuN+/BrdU+ cells. To control for this and to confirm our results, we postnatally electroporated SVZ cells in P1 pups with Eed shRNA or empty control constructs together with pCAG-IRES-GFP (Fig. 3*E*). Five days postelectroporation, GFP immunofluorescence in the SVZ revealed similarly large numbers of electroporated SVZ radial glia-like NSCs in control and Eed KD suggesting similar electroporation efficiency in the 2 groups (Fig. 3*F*). However, at this time point, the density of OB GFP+ cells decreased more than 35% in the knockdown compared with control mice (Fig. 3*G*, *I*). Neither oligodendrocyte differentiation nor cell death accounted for these differences (Figure S3A, S3B and S3C). By 14 dpe, GFP+ cells entered the OB granular layer and displayed mature morphology in controls (Fig. 3*H*). GFP-labeled cells were found in all layers of control OBs 14dpe but were predominantly in the granule layer (Fig. 3*H*). After Eed KD fewer GFP+ cells were seen in all layers. Eed knockdown caused a greater than 60% reduction in GFP+ cell density in the OB taken as a whole (Fig. 3*H*, *I*). The 2 complementary techniques of conditional knockout and shRNA knockdown revealed that Eed promotes postnatal OB neurogenesis.

### Loss of Eed Selectively Derepresses PRC2 Targets in Neural Stem Cells

**Figure 4.**
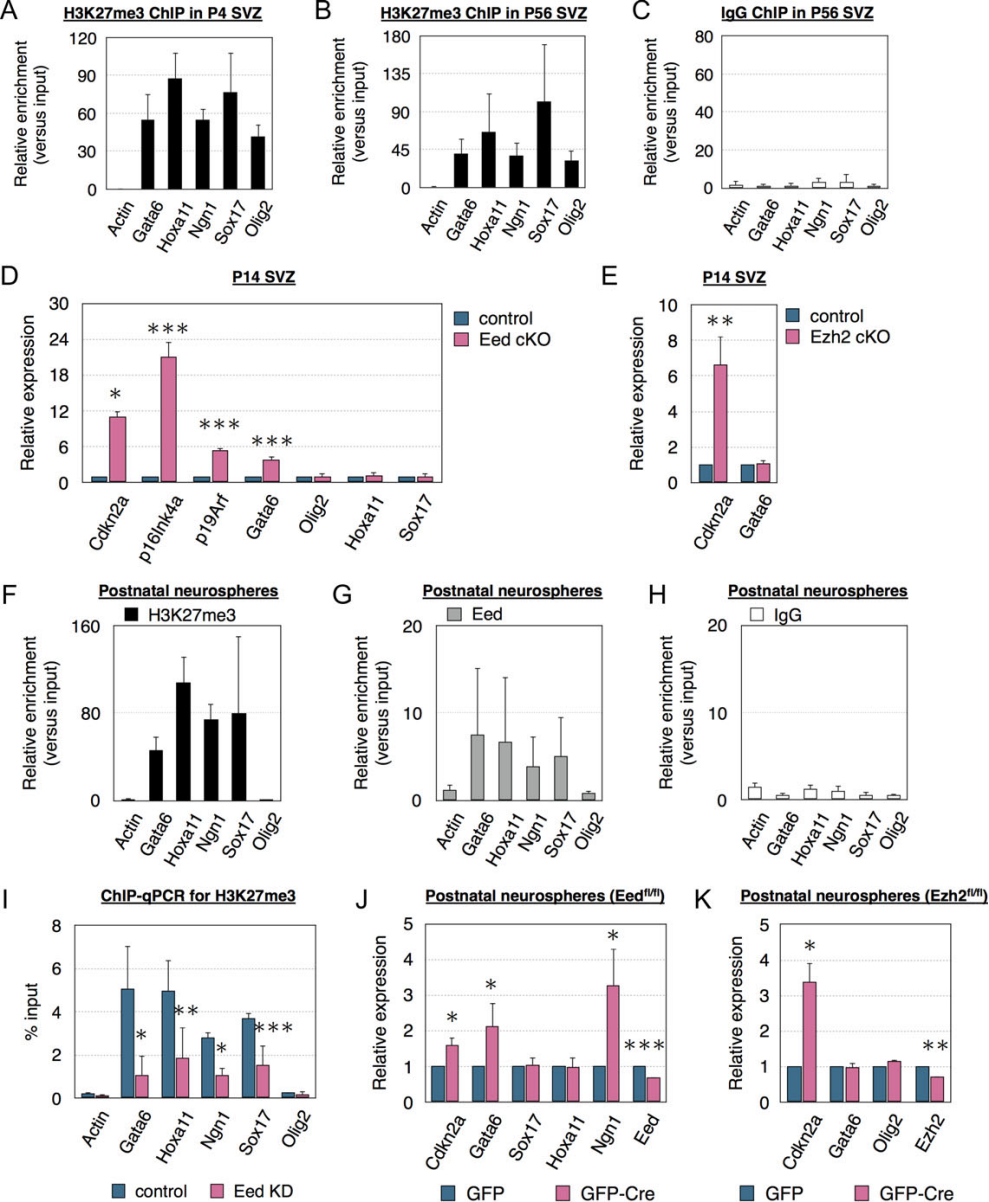
Loss of Eed selectively activates PRC2 targets. (*A*–*C*) ChIP-qPCR from the P4 (*A*) or P56 (*B*) SVZ with anti-H3K27me3 or IgG antibody. *N* = 3. (*D*) qPCR analysis of PRC2 target gene expression in the P14 SVZ of littermate controls and Eed cKO mice. *N* = 3. (*E*) qPCR analysis of PRC2 target gene expression in the P14 SVZ of littermate controls and Ezh2 cKO mice. *N* = 3. (*F*–*H*) ChIP-qPCR using H3K27me3, Eed or IgG antibody of neurospheres cultured from postnatal SVZ. *N* = 3. (*I*) ChIP-qPCR of neurospheres transfected with control or Eed KD constructs using anti-H3K27me3 antibody. *N* = 3. (*J*, *K*) qPCR analysis of PRC2 target gene expression in Eed^fl/fl^ or Ezh2^fl/fl^ neurospheres transfected with GFP or GFP-Cre constructs. *N* = 3. Data are shown as mean ± SD (*A*–*C*, *F*–*I*) or mean ± SEM (*D*, *E*, *J*, *K*) and analyzed by two-tailed Student *t*-test. *P < 0.05, **P < 0.01, ***P < 0.001.

Because Eed and Ezh2 are involved in histone 3 lysine 27 trimethylation (H3K27me3), we determined if the H3K27me3 repressive mark was associated with PRC2 targets in the SVZ. Chromatin immunoprecipitation (ChIP) analysis of P4 and P56 SVZ showed H3K27me3 enrichment on multiple classic PRC2 targets, including Gata6, Hoxa11, Ngn1, Sox17, and Olig2 (Fig. 4*A*–*C*). Cdkn2a expresses 2 transcripts, p16Ink4a and p19Arf (Kamijo et al. 1997). PRC2 represses Cdkn2a during development (Pereira et al. 2010; Hidalgo et al. 2012; Xie et al. 2013; Hwang et al. 2014), and both Eed and Ezh2 conditional knockout (Materials and methods and Supplemental results) increased SVZ Cdkn2a expression (Fig. 4*D*, *E*). Previous work showed Ezh2 KO only derepressed p16Ink4a but not p19Arf in the SVZ (Hwang et al. 2014). In contrast, we found, that Eed loss increased both p16Ink4a and p19Arf expression (Fig. 4*D*). Eed cKO only increased expression of Gata6 among other PRC2 targets and CDKIs (Fig. 4*D* and S4A). In contrast Ezh2 cKO did not alter Gata6 expression in the SVZ (Fig. 4*E*).

SVZ heterogeneity implied the above results could have occurred in multiple cell types. We looked for PRC2 and Eed targets in NSC and TAP-enriched self-renewing neurospheres. ChIP analysis revealed H3K27me3 and Eed occupancy on Gata6, Hoxa11, Ngn1, and Sox17 (Fig. 4*F*, *G*, *H*), similar to the SVZ. Eed KD correspondingly decreased H3K27 occupancy on these targets (Fig. 4*I*). Neurospheres cultured from Eed^fl/fl^ or Ezh2^fl/fl^ pups were nucleofected with either GFP control or GFP-Cre plasmids, at 40–50% efficiency causing a 30–40% decrease in RNA (Fig. 4*J*, *K*). Gata6 expression increased more than 2-fold in the GFP-Cre neurospheres 7 days post transfection (Fig. 4*J*). Expression was selectively increased, Gata6, Cdkn2a, and Ngn1 increased but Sox17 and Hoxa11 did not (Fig. 4*J*). In contrast to Eed cKO, Ezh2 cKO in vitro only increased expression of Cdkn2a but did not affect Gata6 or other PRC2 targets (Fig. 4*K*). Our in vivo and in vitro data indicate Eed selectively suppresses Gata6 in contrast to Ezh2, which did not affect expression of this transcription factor.

### Gata6 Overexpression Limits Neurogenesis Both In Vitro and In Vivo

**Figure 5.**
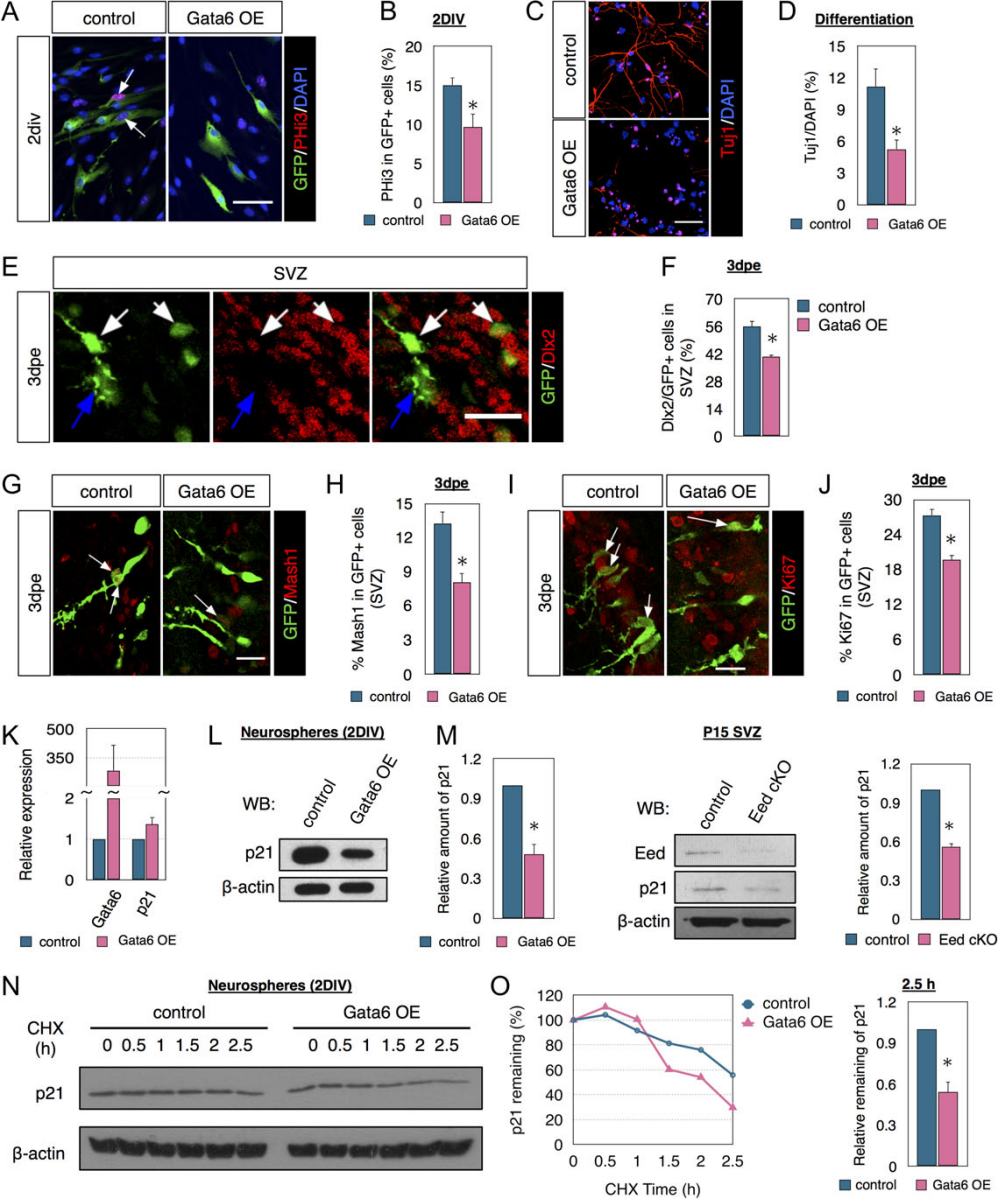
Eed and Gata6 fine-tune p21 protein levels for neurogenesis. (*A*, *B*) Immunostaining and quantification of PHi3 in GFP+ cells derived from neurospheres transfected with either GFP or Gata6 cDNA. The arrows indicate PHi3+/GFP+ cells. *N* = 3. (*C*, *D*) Immunostaining and quantification of Tuj1 in differentiating cells from 7 DIV neurospheres transfected with either GFP or Gata6 cDNA. *N* = 3. (*E*, *F*) Immunocytochemistry and quantification of Dlx2 in GFP+ cells in SVZ of pups electroporated with either GFP or Gata6 cDNA. *N* = 3. (*G*, *H*) Immunostaining and quantification of Mash1 in GFP+ cells in SVZ of pups electroporated with either GFP or Gata6 cDNA. The arrows indicate Mash1+/GFP+ cells. *N* = 3. (*I*, *J*) Immunostaining and quantification of Ki67+in GFP+ cells in SVZ of pups electroporated with either GFP or Gata6 cDNA. The arrow indicates Ki67+/GFP+ cells. *N* = 3. (*K*, *L*) qPCR (*K*) and western blot (*L*) analysis of p21 expression in neurospheres transfected with either GFP or Gata6 cDNA. *N* = 3. (*M*) Western blot analysis of p21 in P15 SVZ tissues from littermate control or Eed cKO mice. *N* = 3. (*N*, *O*) p21 protein stability time course in neurospheres transfected with either GFP or Gata6 cDNA and treated with cycloheximide (CHX) added 48 h after nucleofection. *N* = 3. Data are shown as mean ± SEM and analyzed by two-tailed Student *t*-test. *P < 0.05. Scale bars represent 50 μm in (*A*, *C*); and 20 μm in (*E*, *G*, *H*).

*GATA6* is suppressed via CpG island hypermethylation in glioblastoma (Martinez et al. 2009; Skiriute et al. 2012), suggesting it normally limits proliferation. The role of Gata6 in neural development was unknown, but our results also suggested it limits mitosis. Gata6 overexpression (OE) in cultured NSCs reduced the percent of nucleofected (GFP+) cells that were PHi3+ (M phase of cell cycle) by 30% (Fig. 5*A*, *B*). Gata6 OE did not alter Caspase-3+/GFP+ cell numbers in vitro (Fig. S4B), suggesting no effect on cell death. Gata6 OE also had no effect on nestin or GFAP immunostaining at 2DIV (Fig. S4C, D, E). In differentiation assays without growth factors Gata6 OE decreased the percentage of nucleofected cells that were Tuj1+ at 7DIV, and also decreased neurite length (Fig. 5*C*, *D*), together suggesting a negative effect on neurogenesis and/or neuronal differentiation. In contrast, differentiation assays showed the percent of cells that expressed GFAP after Gata6 OE was not significantly different compared with controls (Fig. S4F), suggesting Gata6 does not affect fate choice in SVZ NSC.

To confirm that Gata6 limits SVZ neurogenesis, we coelectroporated Gata6 cDNA with GFP in ~P1 pups. The majority of SVZ cells expressing Mash1 and Dlx2 are TAPs (Mamber et al. 2013). At 3dpe, fewer GFP+ cells in the Gata6 OE group expressed Dlx2 (Fig. 5*E*, *F*) or Mash1 (Fig. 5*G*, *H*) in the SVZ. Of note, neither Gata6 OE nor the loss of Eed directly affected Mash1 transcription (Fig. S4G, H) suggesting they inhibited lineage progression from NSC to TAPS rather than reducing marker expression. Similar to the reduction of proliferation in vitro, Gata6 OE reduced the percentage of electroporated cells that expressed Ki67 (Fig. 5*I*, *J*). These novel results show the transcription factor Gata6 is sufficient to restrict proliferation and neurogenesis in SVZ cells.

### Elevated Gata6 Reduces p21 Protein Stability

p21 maintains SVZ NSCs (Kippin et al. 2005; Marques-Torrejon et al. 2013; Porlan et al. 2013), so we hypothesized that high levels of Gata6 expression limit SVZ neurogenesis through p21. Gata6 OE in vitro did not significantly alter p21 mRNA expression (Fig. 5*K*), but p21 protein levels were decreased (Fig. 5*L*). Eed cKO decreased p21 protein in vivo, further suggesting functional interaction with Gata6 (Fig. 5*M*). MicroRNA clusters (miR-224, miR-291, miR-303b, etc.) and RNA binding proteins (Msi1, Fxr1, etc.) regulate p21 expression post-transcriptionally (Dolezalova et al. 2012; Freeman and Espinosa 2013), but Gata6 OE in neurospheres did not alter miRNA (8 examined) and RNA binding protein expression (11 examined) (Fig. S5A, B), suggesting they do not participate in this context. Stabilization and degradation of p21 protein is important in tumorigenesis (Abbas and Dutta 2009) and to determine protein stability, we examined p21 levels in neurospheres treated with the protein synthesis inhibitor (CHX). Fortyeight hrs after nucleofection, cells were treated with CHX and analyzed 0–2.5 h later. Gata6 OE significantly decreased p21’s half life after CHX treatment (Fig. 5*N*, *O*). The protease inhibitor MG132, increased p21 levels relatively more in Gata6 OE than control cells (Fig. S5C) suggesting Gata6 regulates p21 protein stability.

### Eed Regulates SVZ Neurogenesis Partially Through Gata6 and p21

**Figure 6.**
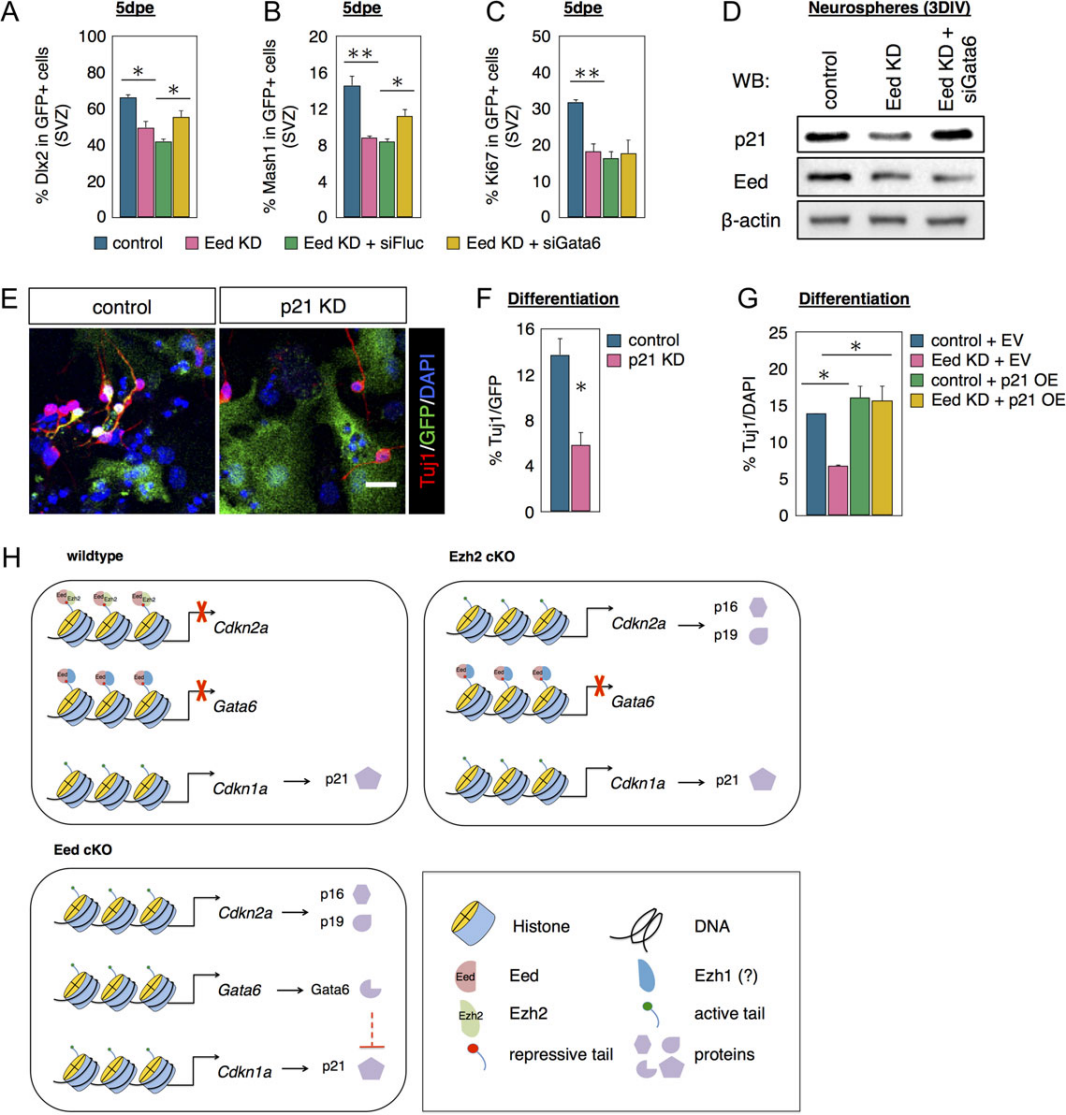
Gata6 knockdown and p21 overexpression partially rescues Eed loss in NSCs. (*A*–*C*) Quantifications of Dlx2 (*A*) or Mash1 (*B*) or Ki67 (*C*) in GFP+ cells in SVZ of pups electroporated with different plasmids as listed in the figure. *N* = 3–5. (*D*) Western blot analysis of p21 in neurospheres after Eed and Gata6 double knockdown. (*E*, *F*) Immunostaining and quantification of β-III tubulin as detected with the Tuj1 antibody in NSC derived from neurospheres transfected with either empty vector or p21 shRNA. *N* = 3. (*G*) Quantification of Tuj1+ cells in NSC derived from neurospheres transfected with control, Eed KD, empty vector (EV), or p21 OE plasmids as listed. *N* = 3. (*H*) A schematic diagram of molecular pathways regulated by Eed and Ezh2 in NSCs. Data are shown as mean ± SEM and analyzed by two-tailed Student *t*-test or one way ANOVA. *P < 0.05, **P < 0.01. Scale bar represents 20 μm in (*E*).

If Gata6 is necessary for Eed regulation of SVZ neurogenesis, Gata6 repression should rescue neurogenesis deficits caused by Eed loss. To test this, P1 pups were electroporated with an Eed shRNA construct alone or along with either a luciferase siRNA (siFluc) as a negative control or a Gata6 siRNA that was confirmed in vitro (Fig. S5D). The percentage of GFP+ cells that were Dlx2+, Mash1+, or Ki67+ was comparable between the Eed shRNA and Eed shRNA plus siFluc groups (Fig. 6*A*–*C*), establishing a baseline for siGata6 comparison. 5dpe the percentage of electroporated GFP+ cells expressing Dlx2 decreased after Eed KD (Fig. 6*A*). When pups were co-electroporated with Eed shRNA and siGata6 constructs, the percentage of GFP+ cells that were Dlx2+ increased statistically compared with the siFluc group showing a partial but significant rescue (Fig. 6*A*). Similar to Dlx2, the percent of electroporated GFP+ cells expressing Mash1 decreased in the Eed shRNA group compared with controls (Fig. 6*B*). When pups were coelectroporated with Eed shRNA and siGata6 constructs, the percent of GFP+ cells that were Mash1+ increased significantly by 35% (Fig. 6*B*). Since we showed that Eed orchestrates p16/p19 and p21 cell cycle inhibitor expression in the SVZ (Figs 4 and 5), we examined whether Gata6 KD could rescue the decreased proliferation induced by Eed KD. Confirming our cKO results, Eed KD decreased the percent of electroporated GFP+ cells that were Ki67+ by 43% (Fig. 6*C*). However, double knockdown of Eed and Gata6, did not increase the percent of GFP+/Ki67+ suggesting Gata6 KD was insufficient to rescue proliferation after Eed KD.

We next confirmed Gata6 is necessary for Eed loss-of-function induced P21 protein decrease. Eed KD in neurospheres reduced p21 protein, confirming our Eed cKO effects (Fig. 6*D*). Eed and Gata6 double KD in neurospheres restored p21 levels (Fig. 6*D*) indicating Gata6 was indeed necessary for the effect of Eed loss on P21. To confirm if Eed loss decreases neurogenesis via p21, we carried out p21 KD in neurospheres. p21 KD reduced the percentage of nucleofected cells expressing β-III tubulin compared with controls (Fig. 6*E*, *F*), which was consistent with the literature (Porlan et al. 2013) and suggested reduced neurogenesis and/or differentiation. Moreover, p21 OE rescued the percent of β-III tubulin+ cells in Eed KD of NSC cultures (Fig. 6*G*). These data establish a mechanism by which the PRC2/Eed complex regulates Gata6 and p21 to refine neurogenic competence in NSCs, as schematized in Figure 6*H*.

### Eed is Dispensable for Parenchymal Reactive Astrocyte Proliferation After Injury but Necessary for Injury-Induced SVZ Activation

**Figure 7.**
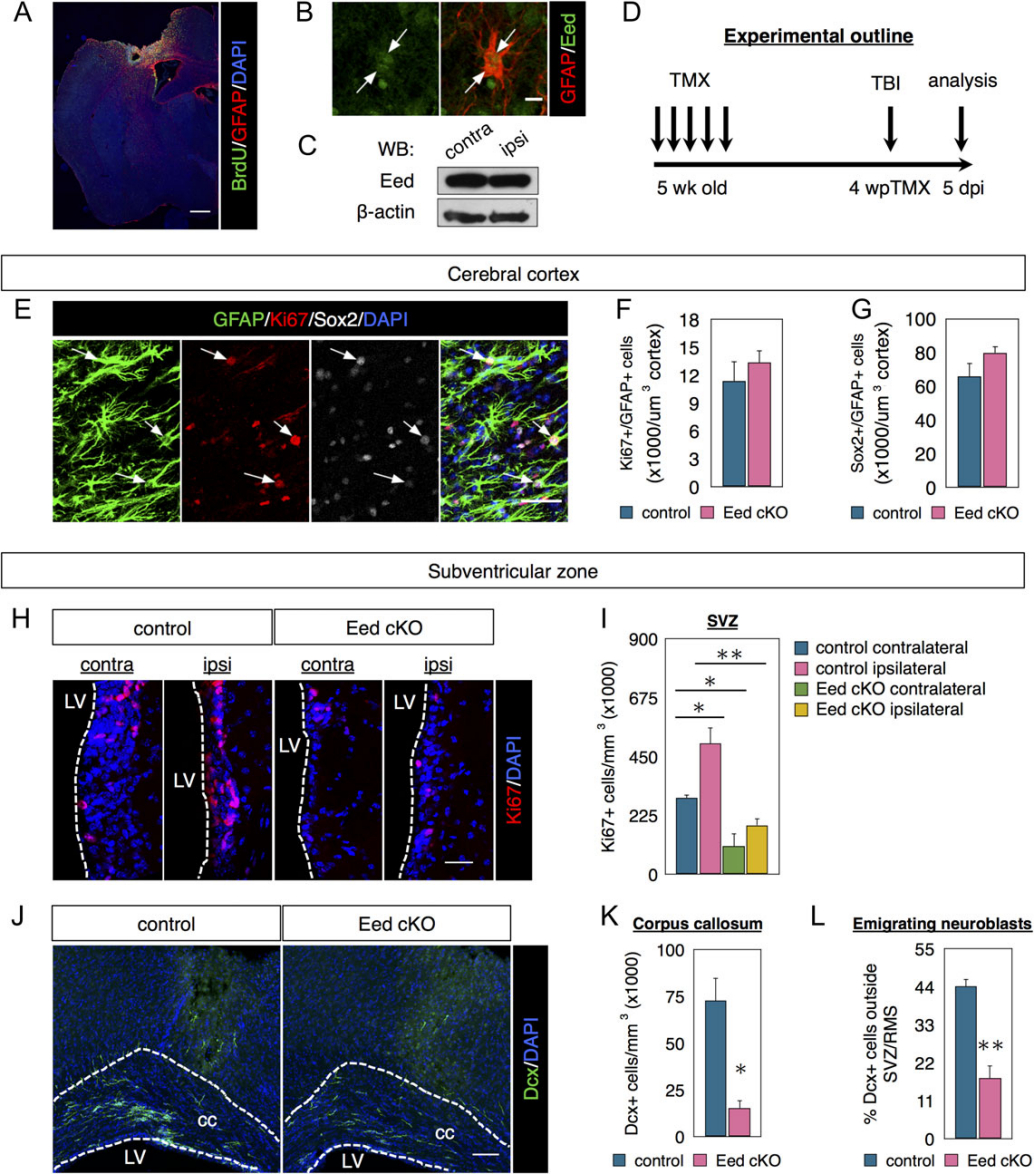
Eed is required for injury-induced SVZ proliferation. (*A*) Coimmunostaining of GFAP and BrdU in the cortical lesion. (*B*) Coimmunostaining of GFAP and Eed in reactive astrocytes in the injured cerebral cortex. Arrows indicate astrocytes undergoing cell division. (*C*) Western blot of Eed expression in the ipsilateral and contralateral cerebral cortex. (*D*) Timeline of experimental design. (*E*–*G*) Coimmunostaining and quantifications of GFAP, Sox2, and Ki67 in the injured cerebral cortex. *N* = 3. (*H*, *I*) Immunostaining and quantifications of Ki67 in the SVZ. *N* = 3. (*J*–*L*) Immunostaining and quantifications of Dcx in the corpus callosum. *N* = 3. Data are shown as mean ± SEM and analyzed by two-tailed Student *t*-test or one way ANOVA. *P < 0.05, **P < 0.01. Scale bars represent 500 μm in (*A*), 10 μm in (*B*), 50 μm in (*E*), 30 μm in (*H*), and 100 μm in (*J*).

Parenchymal astrocytes are generally postmitotic, but can become reactive after injury and become neurogenic (Buffo et al. 2008; Sirko et al. 2013). We tested Eed necessity for parenchymal astrocyte proliferation after a cerebral cortex injury (Fig. 7*A*) that activates SVZ neurogenesis and neuroblast emigration (Szele and Chesselet 1996; Sundholm-Peters et al. 2005). Peri-lesion GFAP+ reactive astrocytes were Eed+ (Fig. 7*B*) but overall Eed levels were similar in ipsi- and contralateral cortex (Fig. 7*C*). To test Eed necessity for cortical astrogenesis after injury, we compared 5-week old Glast-CreERT; Eed^fl/fl^ (cKO) with Eed^fl/fl^ (control) mice. Both groups received tamoxifen and then cortical lesions 4 weeks later (Fig. 7*D*). Peri-lesion astrocytes expressed high levels of GFAP and Ki67 and many expressed Sox2 (Fig. 7*E*). Control and cKO mice had similar numbers of Ki67+/GFAP+ proliferative astrocytes and Sox2+/GFAP+ cells (Fig. 7*F*, *G*), suggesting Eed is dispensable for parenchymal astrocytic proliferation.

To determine if Eed mediates SVZ function after injury, increasing proliferation, we compared Eed cKOs with controls. As expected, the SVZ ipsilateral to the lesion contained more proliferative cells (Ki67+) than the contralateral SVZ in control mice 5 days post injury (Fig. 7*H*, *I*). After Eed loss, both ipsilateral and contralateral SVZs had significantly fewer Ki67+ cells than controls. More importantly, cell proliferation was not statistically significantly increased after Eed cKO in the ipsilateral SVZ (Fig. 7*H*, *I*). Caspase-3 immunohistochemistry indicated this was not due to enhanced cell death (data not shown). SVZ neuroblasts migrate to the OB, but cortical injury redirects some into the corpus callosum and cortex (Goings et al. 2004; Saha et al. 2013). Fewer neuroblasts were found in the corpus callosum in Eed cKO compared with control mice (Fig. 7*J*, *K*) consistent with the proliferation deficiency. After injury approximately 20% of all Dcx+ cells were found in the CC in Eed cKO compared with 44% in controls, suggesting Eed necessity for a robust response to cortical injury (Fig. 7*L*).

## Discussion

The most important finding here is that Eed loss significantly reduces SVZ NSC activation and neurogenesis. It does so by increasing Gata6 expression, which reduces p21 protein levels. We confirmed these findings in vivo and in vitro with multiple knockdown, overexpression and rescue studies. Gata6, however, is not affected by Ezh2, which suggests PRC2 works via multiple distinct molecular pathways. Eed is also necessary for SVZ proliferation but not for parenchymal astrocytosis.

### Eed Regulates SVZ NSC Proliferation and Neurogenesis via Gata6 and p21

Several experiments demonstrated Eed loss reduces SVZ NSC proliferation and neurogenesis. We crossed floxed Eed (Xie et al. 2013) and Glast-CreERT2 mice (Mori et al. 2006) to knockout Eed from SVZ NSC and their progeny. Though most NSC have Eed deleted after Tamoxifen, a few NSC likely “escaped” Cre recombination and thus retained normal Eed levels. This was consistent with incomplete loss of H3K27me3 in LRC-BrdU cells. Additionally, the SVZ is heterogeneous and some SVZ neural stem cells may not express GLAST. Nevertheless, conditional knockout of Eed significantly decreased the percent of proliferating SVZ NSCs. Interestingly, Eed conditional knockout also caused more GFAP+ and label-retaining cells to express S100β. RNA-Seq showed quiescent SVZ NSCs express >200-fold more S100β than active NSCs (Codega et al. 2014). The percentage of SVZ LRCs that express S100β significantly increased after Eed cKO, thus SVZ LRCs are likely to become quiescent after Eed loss. Finally, Eed conditional knockout and knockdown reduced newborn neuronal numbers in the OB suggesting reduced neurogenesis. We believe that the reduction in newborn neuroblasts in the OB after Eed loss is because fewer NSC become activated thereby inhibiting lineage progression to neuroblasts. Alternatively, Eed/Gata6/p21 may function directly in neuroblasts to reduce their numbers in the OB. Which stage of SVZ lineage does Eed affect? Eed knockdown with electroporation initially targets NSC (Boutin et al. 2008). Eed is expressed in all SVZ cells, and Eed and H3K27me3 expression were confirmed in label-retaining NSC, GFAP+/EGFR+NSC, proliferative TAPs, and Dcx+ neuroblasts. This is consistent with Eed affecting NSC and then also functioning at subsequent steps in the SVZ lineage.

Eed loss increased Gata6 expression. Gata6 is a transcription factor essential for heart and primitive gut development (Burch 2005). Gata6’s role in mammalian neurogenesis was unknown but hypermethylation represses Gata6 in glioblastomas (Martinez et al. 2009; Skiriute et al. 2012), suggesting Gata6 normally limits proliferation. Accordingly, Gata6 OE decreased SVZ proliferation in vivo and in vitro. Further studies showed Gata6 OE decreased TAP and neuroblast marker-positive cells. Gata6 OE could have increased Gata6 more than in Eed cKO and not reflected true physiological fluctuations. However, Gata6 OE may have a “ceiling effect”; the response to increased expression may have a threshold. These caveats aside, Gata6 knockdown suggest it is necessary for reduced neurogenesis after Eed loss—it partially rescued Eed loss effects. p16/p19 expression increased after Eed loss and this may have restricted general proliferation, limiting the extent of the rescue.

Eed loss-of-function increased p16/p19 (Cdkn2a) but did not significantly alter p21 (Cdkn1a) mRNA levels. In contrast, Eed cKO in vivo and KD in vitro reduced p21 protein levels. Similarly, Gata6 OE reduced p21 protein levels. MicroRNAs and RNA binding proteins modulate p21 expression predicting they are Gata6 transcriptional targets. However, Gata6 OE did not alter microRNA and RNA binding protein expression suggesting they do not affect p21 stabilization; therefore, how Gata6 destabilizes p21 is unclear. Nonetheless, Gata6 was required for Eed KD to decrease p21 protein levels since Gata6 loss blocked the effect. As well, Eed KD and p21 KD similarly reduced the number of neuroblast marker-positive cells and p21 OE rescued the Eed KD effect.

Thus, Eed normally represses Gata6, which maintains p21 protein. Together with Eed’s effect on p16/p19 this mechanism regulates SVZ NSC maintenance, proliferation and neurogenesis. Since post-transcriptional regulatory processes fine-tune p21 protein levels, p21 may have dose-dependent roles in the SVZ. Most knowledge of p21 function in the SVZ is from constitutive knockouts (Kippin et al. 2005; Marques-Torrejon et al. 2013; Porlan et al. 2013). Nonmutant approaches, such as p21 promoter modulation, and determination of p21 protein levels in stem cell lineages will clarify its functions.

### Eed is Necessary for Injury-Induced SVZ Activation but Dispensable for Parenchymal Astrocytosis

Increased SVZ proliferation and progenitor emigration towards lesions can improve histological and behavioral outcomes (Jin et al. 2010; Young et al. 2011). Brain injury induces both SVZ astrocyte-like NSC proliferation and parenchymal astrocytosis but the overlap of molecular mechanisms regulating these two processes is unclear. Since epigenetic mechanisms coordinate astrocytic and NSC-mediated brain repair, we examined Eed function after cortical injury. Eed loss inhibited lesion-induced increases in SVZ proliferation and the number of Dcx+ cells between the SVZ and the injury, showing Eed participates in injury-induced SVZ proliferation. As of now, it is unclear if the post injury effects of Eed on SVZ proliferation are Gata6 or p21 dependent. In contrast to its effects in the SVZ, Eed loss did not alter parenchymal astrocytosis after injury, suggesting Eed is dispensable for this process. Our data reveals a fundamental difference in post injury molecular regulation in SVZ astrocyte-like NSCs compared with parenchymal astrocytes.

### Eed Versus Ezh2—Distinct Cellular Expression and Molecular Regulation

Eed was expressed by label-retaining cells and GFAP+/EGFR+SVZ cells, implying NSC expression (Mamber et al. 2013). In contrast, SVZ label-retaining cells and GFAP+/Sox2+SVZ cells did not exhibit Ezh2 immunofluorescence indicating it was not expressed by NSC (Mamber et al. 2013). However, Ezh2 may be expressed by NSC below the level of detection. Notably Eed, but not Ezh2, loss increased Gata6 expression. Eed and Ezh2 both repress p16/p19 (Cdkn2a)—our work is concordant with Hwang et al., 2014, showing Ezh2 loss increased p16. Eed and Ezh2 loss-of-function may regulate other distinct genes through selective H3K27me3 targets. Future RNA-sequencing studies could establish these global differences and help place Gata6 and p21 in the context of broad gene regulatory networks.

## Conclusion

We show Eed is necessary for stem cell activation and neurogenesis. We delineated a functional role for Eed in regulating Gata6 and thereby maintaining p21 protein levels. Interestingly the PRC2 methyltransferase Ezh2 did not alter Gata6 levels suggesting divergence of Eed and Ezh2 function in the SVZ niche. Our study identifies Eed, Gata6, and p21 as possible molecular targets to manipulate stem cell cycles in injury and cancer.

## Supplementary Material

Supplementary Data

## References

[bhx289C1] Abbas T, Dutta A 2009 p21 in cancer: intricate networks and multiple activities. Nat Rev Cancer. 9:400–414. 19440234 10.1038/nrc2657 PMC2722839

[bhx289C2] Bardella C, Al-Dalahmah O, Krell D, Brazauskas P, Al-Qahtani K, Tomkova M, Adam J, Serres S, Lockstone H, Freeman-Mills L, et al 2016 Expression of Idh1R132H in the murine subventricular zone stem cell niche recapitulates features of early gliomagenesis. Cancer Cell. 30:578–594. 27693047 10.1016/j.ccell.2016.08.017 PMC5064912

[bhx289C3] Boutin C, Diestel S, Desoeuvre A, Tiveron MC, Cremer H 2008 Efficient in vivo electroporation of the postnatal rodent forebrain. PLoS One. 3:e1883. 18382666 10.1371/journal.pone.0001883 PMC2270900

[bhx289C4] Buffo A, Rite I, Tripathi P, Lepier A, Colak D, Horn AP, Mori T, Gotz M 2008 Origin and progeny of reactive gliosis: a source of multipotent cells in the injured brain. Proc Natl Acad Sci USA. 105:3581–3586. 18299565 10.1073/pnas.0709002105 PMC2265175

[bhx289C5] Burch JBE 2005 Regulation of GATA gene expression during vertebrate development. Semin Cell Dev Biol. 16:71–81. 15659342 10.1016/j.semcdb.2004.10.002

[bhx289C6] Chesler AT, Le Pichon CE, Brann JH, Araneda RC, Zou DJ, Firestein S 2008 Selective gene expression by postnatal electroporation during olfactory interneuron nurogenesis. Plos One. 3:e1517. 18231603 10.1371/journal.pone.0001517 PMC2204067

[bhx289C7] Cheung TH, Rando TA 2013 Molecular regulation of stem cell quiescence. Nat Rev Mol Cell Biol. 14:329–340. 23698583 10.1038/nrm3591 PMC3808888

[bhx289C8] Codega P, Silva-Vargas V, Paul A, Maldonado-Soto AR, Deleo AM, Pastrana E, Doetsch F 2014 Prospective identification and purification of quiescent adult neural stem cells from their in vivo niche. Neuron. 82:545–559. 24811379 10.1016/j.neuron.2014.02.039 PMC4360885

[bhx289C9] Cooper S, Brockdorff N 2013 Genome-wide shRNA screening to identify factors mediating Gata6 repression in mouse embryonic stem cells. Development. 140:4110–4115. 24046324 10.1242/dev.094615 PMC3775421

[bhx289C10] Doetsch F, Caille I, Lim DA, Garcia-Verdugo JM, Alvarez-Buylla A 1999 Subventricular zone astrocytes are neural stem cells in the adult mammalian brain. Cell. 97:703–716. 10380923 10.1016/s0092-8674(00)80783-7

[bhx289C11] Dolezalova D, Mraz M, Barta T, Plevova K, Vinarsky V, Holubcova Z, Jaros J, Dvorak P, Pospisilova S, Hampl A 2012 MicroRNAs regulate p21(Waf1/Cip1) protein expression and the DNA damage response in human embryonic stem cells. Stem Cells. 30:1362–1372. 22511267 10.1002/stem.1108

[bhx289C12] Fasano CA, Dimos JT, Ivanova NB, Lowry N, Lemischka IR, Temple S 2007 shRNA knockdown of Bmi-1 reveals a critical role for p21-Rb pathway in NSC self-renewal during development. Cell Stem Cell. 1:87–99. 18371338 10.1016/j.stem.2007.04.001

[bhx289C13] Freeman JA, Espinosa JM 2013 The impact of post-transcriptional regulation in the p53 network. Brief Funct Genomics. 12:46–57. 23242178 10.1093/bfgp/els058 PMC3548162

[bhx289C14] Goings GE, Sahni V, Szele FG 2004 Migration patterns of subventricular zone cells in adult mice change after cerebral cortex injury. Brain Res. 996:213–226. 14697499 10.1016/j.brainres.2003.10.034

[bhx289C15] Goings GE, Wibisono BL, Szele FG 2002 Cerebral cortex lesions decrease the number of bromodeoxyuridine-positive subventricular zone cells in mice. Neurosci Lett. 329:161–164. 12165402 10.1016/s0304-3940(02)00611-0

[bhx289C16] Hidalgo I, Herrera-Merchan A, Ligos JM, Carramolino L, Nunez J, Martinez F, Dominguez O, Torres M, Gonzalez S 2012 Ezh1 is required for hematopoietic stem cell maintenance and prevents senescence-like cell cycle arrest. Cell Stem Cell. 11:649–662. 23122289 10.1016/j.stem.2012.08.001

[bhx289C17] Hwang WW, Salinas RD, Siu JJ, Kelley KW, Delgado RN, Paredes MF, Alvarez-Buylla A, Oldham MC, Lim DA 2014 Distinct and separable roles for EZH2 in neurogenic astroglia. eLife. 3:e02439. 24867641 10.7554/eLife.02439 PMC4032491

[bhx289C18] Ihrie RA, Alvarez-Buylla A 2011 Lake-front property: a unique germinal niche by the lateral ventricles of the adult brain. Neuron. 70:674–686. 21609824 10.1016/j.neuron.2011.05.004 PMC3346178

[bhx289C19] Jin K, Wang X, Xie L, Mao XO, Greenberg DA 2010 Transgenic ablation of doublecortin-expressing cells suppresses adult neurogenesis and worsens stroke outcome in mice. Proc Natl Acad Sci USA. 107:7993–7998. 20385829 10.1073/pnas.1000154107 PMC2867852

[bhx289C20] Kamijo T, Zindy F, Roussel MF, Quelle DE, Downing JR, Ashmun RA, Grosveld G, Sherr CJ 1997 Tumor suppression at the mouse INK4a locus mediated by the alternative reading frame product p19ARF. Cell. 91:649–659. 9393858 10.1016/s0092-8674(00)80452-3

[bhx289C21] Kippin TE, Martens DJ, van der Kooy D 2005 p21 loss compromises the relative quiescence of forebrain stem cell proliferation leading to exhaustion of their proliferation capacity. Genes Dev. 19:756–767. 15769947 10.1101/gad.1272305 PMC1065728

[bhx289C22] Liang Q, De Windt LJ, Witt SA, Kimball TR, Markham BE, Molkentin JD 2001 The transcription factors GATA4 and GATA6 regulate cardiomyocyte hypertrophy in vitro and in vivo. J Biol Chem. 276:30245–30253. 11356841 10.1074/jbc.M102174200

[bhx289C23] Llorens-Bobadilla E, Zhao S, Baser A, Saiz-Castro G, Zwadlo K, Martin-Villalba A 2015 Single-cell transcriptomics reveals a population of dormant neural stem cells that become activated upon brain injury. Cell Stem Cell. 17:329–340. 26235341 10.1016/j.stem.2015.07.002

[bhx289C24] Mamber C, Kozareva DA, Kamphuis W, Hol EM 2013 Shades of gray: the delineation of marker expression within the adult rodent subventricular zone. Prog Neurobiol. 111:1–16. 23994259 10.1016/j.pneurobio.2013.07.003

[bhx289C25] Margueron R, Reinberg D 2011 The Polycomb complex PRC2 and its mark in life. Nature. 469:343–349. 21248841 10.1038/nature09784 PMC3760771

[bhx289C26] Marques-Torrejon MA, Porlan E, Banito A, Gomez-Ibarlucea E, Lopez-Contreras AJ, Fernandez-Capetillo O, Vidal A, Gil J, Torres J, Farinas I 2013 Cyclin-dependent kinase inhibitor p21 controls adult neural stem cell expansion by regulating Sox2 gene expression. Cell Stem Cell. 12:88–100. 23260487 10.1016/j.stem.2012.12.001 PMC3714747

[bhx289C27] Martinez R, Martin-Subero JI, Rohde V, Kirsch M, Alaminos M, Fernandez AF, Ropero S, Schackert G, Esteller M 2009 A microarray-based DNA methylation study of glioblastoma multiforme. Epigenetics. 4:255–264. 19550145 10.4161/epi.9130

[bhx289C28] Matsuda T, Cepko CL 2004 Electroporation and RNA interference in the rodent retina in vivo and in vitro. Proc Natl Acad Sci USA. 101:16–22. 14603031 10.1073/pnas.2235688100 PMC314130

[bhx289C29] Molofsky AV, Pardal R, Iwashita T, Park IK, Clarke MF, Morrison SJ 2003 Bmi-1 dependence distinguishes neural stem cell self-renewal from progenitor proliferation. Nature. 425:962–967. 14574365 10.1038/nature02060 PMC2614897

[bhx289C30] Mori T, Tanaka K, Buffo A, Wurst W, Kuhn R, Gotz M 2006 Inducible gene deletion in astroglia and radial glia—a valuable tool for functional and lineage analysis. Glia. 54:21–34. 16652340 10.1002/glia.20350

[bhx289C31] Nishino J, Kim I, Chada K, Morrison SJ 2008 Hmga2 promotes neural stem cell self-renewal in young but not old mice by reducing p16Ink4a and p19Arf expression. Cell. 135:227–239. 18957199 10.1016/j.cell.2008.09.01 PMC2582221

[bhx289C32] Pereira JD, Sansom SN, Smith J, Dobenecker MW, Tarakhovsky A, Livesey FJ 2010 Ezh2, the histone methyltransferase of PRC2, regulates the balance between self-renewal and differentiation in the cerebral cortex. Proc Natl Acad Sci USA. 107:15957–15962. 20798045 10.1073/pnas.1002530107 PMC2936600

[bhx289C33] Pluchino S, Quattrini A, Brambilla E, Gritti A, Salani G, Dina G, Galli R, Del Carro U, Amadio S, Bergami A, et al 2003 Injection of adult neurospheres induces recovery in a chronic model of multiple sclerosis. Nature. 422:688–694. 12700753 10.1038/nature01552

[bhx289C34] Porlan E, Morante-Redolat JM, Marques-Torrejon MA, Andreu-Agullo C, Carneiro C, Gomez-Ibarlucea E, Soto A, Vidal A, Ferron SR, Farinas I 2013 Transcriptional repression of Bmp2 by p21(Waf1/Cip1) links quiescence to neural stem cell maintenance. Nat Neurosci. 16:1567–1575. 24097040 10.1038/nn.3545

[bhx289C35] Raponi E, Agenes F, Delphin C, Assard N, Baudier J, Legraverend C, Deloulme JC 2007 S100B expression defines a state in which GFAP-expressing cells lose their neural stem cell potential and acquire a more mature developmental stage. Glia. 55:165–177. 17078026 10.1002/glia.20445 PMC2739421

[bhx289C36] Saha B, Peron S, Murray K, Jaber M, Gaillard A 2013 Cortical lesion stimulates adult subventricular zone neural progenitor cell proliferation and migration to the site of injury. Stem Cell Res. 11:965–977. 23900166 10.1016/j.scr.2013.06.006

[bhx289C37] Sanai N, Alvarez-Buylla A, Berger MS 2005 Neural stem cells and the origin of gliomas. N Engl J Med. 353:811–822. 16120861 10.1056/NEJMra043666

[bhx289C38] Shen X, Kim W, Fujiwara Y, Simon MD, Liu Y, Mysliwiec MR, Yuan GC, Lee Y, Orkin SH 2009 Jumonji modulates polycomb activity and self-renewal versus differentiation of stem cells. Cell. 139:1303–1314. 20064376 10.1016/j.cell.2009.12.003 PMC2810107

[bhx289C39] Shen X, Liu Y, Hsu YJ, Fujiwara Y, Kim J, Mao X, Yuan GC, Orkin SH 2008 EZH1 mediates methylation on histone H3 lysine 27 and complements EZH2 in maintaining stem cell identity and executing pluripotency. Mol Cell. 32:491–502. 19026780 10.1016/j.molcel.2008.10.016 PMC2630502

[bhx289C40] Sirko S, Behrendt G, Johansson PA, Tripathi P, Costa M, Bek S, Heinrich C, Tiedt S, Colak D, Dichgans M, et al 2013 Reactive glia in the injured brain acquire stem cell properties in response to sonic hedgehog glia. Cell Stem Cell. 12:426–439. 23561443 10.1016/j.stem.2013.01.019

[bhx289C41] Skiriute D, Vaitkiene P, Saferis V, Asmoniene V, Skauminas K, Deltuva VP, Tamasauskas A 2012 MGMT, GATA6, CD81, DR4, and CASP8 gene promoter methylation in glioblastoma. BMC Cancer. 12:218. 22672670 10.1186/1471-2407-12-218 PMC3404983

[bhx289C42] Stewart SA, Dykxhoorn DM, Palliser D, Mizuno H, Yu EY, An DS, Sabatini DM, Chen IS, Hahn WC, Sharp PA, et al 2003 Lentivirus-delivered stable gene silencing by RNAi in primary cells. RNA. 9:493–501. 12649500 10.1261/rna.2192803 PMC1370415

[bhx289C43] Su IH, Basavaraj A, Krutchinsky AN, Hobert O, Ullrich A, Chait BT, Tarakhovsky A 2003 Ezh2 controls B cell development through histone H3 methylation and Igh rearrangement. Nat Immunol. 4:124–131. 12496962 10.1038/ni876

[bhx289C44] Sundholm-Peters NL, Yang HK, Goings GE, Walker AS, Szele FG 2005 Subventricular zone neuroblasts emigrate toward cortical lesions. J Neuropathol Exp Neurol. 64:1089–1100. 16319719 10.1097/01.jnen.0000190066.13312.8f

[bhx289C45] Szele FG, Chesselet MF 1996 Cortical lesions induce an increase in cell number and PSA-NCAM expression in the subventricular zone of adult rats. J Comp Neurol. 368:439–454. 8725350 10.1002/(SICI)1096-9861(19960506)368:3<439::AID-CNE9>3.0.CO;2-6

[bhx289C46] Tavares L, Dimitrova E, Oxley D, Webster J, Poot R, Demmers J, Bezstarosti K, Taylor S, Ura H, Koide H, et al 2012 RYBP-PRC1 complexes mediate H2A ubiquitylation at polycomb target sites independently of PRC2 and H3K27me3. Cell. 148:664–678. 22325148 10.1016/j.cell.2011.12.029 PMC3281992

[bhx289C47] Woodhead GJ, Mutch CA, Olson EC, Chenn A 2006 Cell-autonomous beta-catenin signaling regulates cortical precursor proliferation. J Neurosci. 26:12620–12630. 17135424 10.1523/JNEUROSCI.3180-06.2006 PMC2867669

[bhx289C48] Xie H, Xu J, Hsu JH, Nguyen M, Fujiwara Y, Peng C, Orkin SH 2013 Polycomb repressive complex 2 regulates normal hematopoietic stem cell function in a developmental-stage-specific manner. Cell Stem Cell. 14(1):68–80. 24239285 10.1016/j.stem.2013.10.001 PMC3947409

[bhx289C49] Young CC, Brooks KJ, Buchan AM, Szele FG 2011 Cellular and molecular determinants of stroke-induced changes in subventricular zone cell migration. Antioxid Redox Signal. 14:1877–1888. 20673127 10.1089/ars.2010.3435 PMC3078507

