# Supplementary material for: Polycomb Protein Eed is Required for Neurogenesis and Cortical Injury Activation in the Subventricular Zone
Source: Cereb Cortex. 2018 Feb 3;28(4):1369–82. doi: 10.1093/cercor/bhx289 (PMC6093351; doi:10.1093/cercor/bhx289)
Supplement: Supplementary Data [file bhx289_supplementary-materials.zip › Revised Supplemental results.docx]

**Supplemental results**

**Ezh2 cKO decreases neurogenesis**

We crossed Glast-CreERT2 mice with Ezh2^fl/fl^ mice (Su IH et al. 2003; Mori T et al. 2006) to remove Ezh2 function in postnatal SVZ NSCs via tamoxifen (TMX) administration in P0~P1 pups (Materials and methods). Genotyping and RT-PCR confirmed Ezh2 cKO (Figure S2A and S2B). Ezh2 immunoreactivity was dramatically reduced at P14 in the SVZ of Glast-CreERT2;Ezh2^fl/fl^ mice, (Figure S2C) but we found no obvious change in H3K27me3 staining (data not shown).

We first examined the effect of Ezh2 cKO at P28 (Figure S2D). Ezh2 cKO SVZ had fewer Ki67+ (proliferating cells) and fewer Mash1+ (TAPs) at P28 (Figure S2E and S2F). However the percent of GFAP+/Sox2+ cells that expressed Ki67 was not significantly different after Ezh2 cKO (Figure S2G). Ezh2 CKO mice had slightly weaker Dcx immunostaining in the SVZ (Figure S2H). RT-PCR confirmed significant Mash1 and Dcx decreases at P28 in Ezh2 cKO and Id2 was also reduced (Figure S2I).

We next administered tamoxifen at P0, BrdU from P29 to P33 and analysed neurogenesis at P75 (Figure S2J). Ezh2 loss decreased the number of NeuN+/BrdU+ newborn neurons in OB granular and glomerular layers (Figure S2K, S2L and S2M). Dcx+ neuroblast surface area in the RMS also decreased (data not shown). Caspase-3+ immunoreactivity showed the decreased number of newborn neurons was not due to apoptosis in the RMS/SVZ (data not shown). By P75, the number of PHi3+ (Figure S2N) and Mash1+ (Figure S2O and S2P) SVZ cells in Ezh2 cKOs had returned to control levels. Finally, the total number of LRCs in the SVZ was unaffected in the Ezh2 loss-of-function studies (data not shown), suggesting Ezh2 is dispensable for SVZ NSC maintenance and long-term SVZ NSC label retention. These results are overall consistent with (Hwang WW et al. 2014), which showed loss of Ezh2 function reduces SVZ neurogenesis.

**Supplemental Figure Legends:**

**Figure S1. Generation of conditional knockout mice**

(A-B) High magnification image of Eed, BrdU and Ki67 costaining in label-retaining cells.

(C) Coimmunostaining of H3K27me3 and Dcx in the OB.

(D) Coimmunostaining of Ezh2, PHi3 and Dcx in the P42 SVZ.

(E) Genotyping for Eed transgene. Wildtype band is shorter than 200 bp and the floxed band is longer than 200 bp.

(F) qPCR analysis of SVZ tissue from P14 SVZ to confirm Eed knockout efficiency.

(G) Quantification of PHi3+ cells in P14 SVZ of control and Eed cKO mice. N=3.

(H-I) Immunostaining and quantifications of BrdU and H3K27me3 in mice for BrdU label-retaining analysis. White arrows indicate H3K27me3+/BrdU+ cells, while magenta arrows indicate H3K27m3-/BrdU+ cells. Time course as in Fig. 2F. N=3.

(J) Quantification of the number of label-retaining cells in the SVZ. N=3.

Data are shown as mean ± SEM and analysed by two-tailed Student t-test. **p<0.01. Scale bars represent 20 μm in (A), (B), (D) and (H), and 40 μm in (C).

**Figure S2. Ezh2 is dispensable for SVZ neural stem cell maintenance**

(A) Genotyping for Ezh2 transgene. The floxed band is 736bp and the deleted band is 625bp.

(B) qPCR analysis of SVZ tissue from P14 SVZ to confirm Ezh2 knockout efficiency.

(C) Immunostaining of Ezh2 or H3K27me3 in control or Ezh2 cKO SVZ (P14).

(D) Schematic of experimental outline used in E-J.

(E-G) Quantifications of Ki67+ (E), Mash1+ (F), and percent of GFAP+/Sox2+ cells that are Ki67+ (G) in P14 SVZ of control and Ezh2 cKO mice. N=3-4.

(H) Immunostaining of Dcx in P28 SVZ of control and Ezh2 cKO mice.

(I) qPCR analysis of P14 and P28 SVZ. Relative expression is normalised to littermate controls. N=3.

(J) A schematic diagram of the experimental design used in K-P.

(K-M) Costaining for BrdU and NeuN, and quantifications of double labelled cells in the OB. N=3-4.

(N) Quantification of PHi3+ cells in P75 SVZ. N=3-4.

(O-P) Immunostaining and quantification of Mash1+ cells in P75 SVZ. N=3-4.

Data are shown as mean ± SEM and analysed by two-tailed Student t-test. *p<0.05, ***p<0.001. Scale bars represent 20 μm in (C), 60 μm in (H), (O); and 100 μm in (K).

**Figure S3. Control data for Eed knockdown in vivo**

(A-B) Immunostaining and quantification of Olig2 among GFP+ cells in the RMS (5dpe). The arrows indicate Olig2+/GFP+ cells. N=5-6.

(C) Immunostaining of Caspase-3 among GFP+ cells in the RMS (5dpe). The arrow indicates a Caspase-3+/GFP+ cell. N=5-6.

Data are shown as mean ± SEM and analysed by two-tailed Student t-test. Scale bars represent 20 μm in (A) and (C).

**Figure S4. The molecular regulation of Eed/Gata6 in SVZ NSC**

(A) qPCR analysis of gene expression in P14 SVZ from control and Eed cKO mice. N=3.

(B) Quantification of Caspase-3 among GFP+ NSCs in culture (2 days post nucleofection). N=3.

(C) Immunostaining of Nestin among GFP+ cells in culture after control or Gata6 OE nucleofection.

(D-E) Immunostaining and quantification of GFAP among GFP+ cells in culture. N=3.

(F) Quantification of GFAP in differentiated NSC derived from neurospheres 7 DIV transfected with either GFP or Gata6 cDNA. N=3.

(G) qPCR analysis of Mash1 expression in NSCs 2 days after nucleofection with GFP (control) or Gata6 cDNA. N=3.

(H) qPCR analysis of Mash1 expression in Eed^fl/fl^ NSCs nucleofected with GFP or GFP-Cre. N=3.

Data are shown as mean ± SEM and analysed by two-tailed Student t-test. Scale bars represent 50 μm in (C), (D).

**Figure S5. Gata6 overexpression regulates p21 protein stability**

(A-B) qPCR analysis of miRNAs and RBPs in NSCs transfected with GFP or Gata6 cDNA (2 days in culture). N=3.

(C) Western blot of p21 in neurospheres transfected with either control GFP or Gata6 cDNA and treated with MG132 for 4hrs. N=3.

(D) Western blot to validate the knockdown efficiency of siGata6 in NSCs in vitro.

Data are shown as mean ± SEM and analysed by two-tailed Student t-test.

**Supplemental Table 1. Antibodies for immunohistochemistry**

| Antigen | Dilution | Host | Manufacturer Cat. |
| --- | --- | --- | --- |
| BrdU | 1:400 | mouse | Dako M0744 |
| BrdU | 1:500 | sheep | Abcam Ab1893 |
| Caspase-3 | 1:1000 | rabbit | Cell Signaling 9661 |
| Dcx | 1:100 | goat | Santa Cruz SC-8066 |
| Dlx2 | 1:500 | rabbit | Millipore AB5726 |
| Eed | 1:100 | mouse | Abcam ab126542 |
| EGFR | 1:200 | rabbit | Santa Cruz sc-03 |
| Ezh2 | 1:100 | mouse | BD Biosciences 612666 |
| GFAP | 1:400 | rat | Invitrogen 130300 |
| GFAP | 1:5000 | chicken | Abcam ab4674 |
| GFP | 1:500 | chicken | Aves GFP-1010 |
| H3K27me3 | 1:250 | rabbit | Millipore 07-449 |
| Ki67 | 1:500 | rabbit | Abcam ab16667 |
| Mash1 | 1:100 | mouse | BD Biosciences 556604 |
| Nestin | 1:200 | mouse | Millipore MAB353 |
| NeuN | 1:400 | mouse | Millipore MAB377 |
| Olig2 | 1:1000 | rabbit | Millipore AB9610 |
| PHi3 | 1:400 | rabbit | Millipore 06-570 |
| S100β | 1:200 | rabbit | Dako Z031129-2 |
| S100β | 1:100 | mouse | Sigma S2657 |
| Sox2 | 1:500 | rabbit | Millipore AB5603 |
| Sox2 | 1:200 | goat | R&D systems AF2018 |

**Supplemental Table 2. Primers for RT-PCR**

| **Gene** | Sequence (5'-3') |
| --- | --- |
| ***Gata6*** | CTTGCGGGCTCTATATGAAACTCCAT |
|  | TAGAAGAAGAGGAAGTAGGAGTCATAGGGACA |
| ***Sox17*** | GCCAAAGACGAACGCAAGCG |
|  | TTCTCTGCCAAGGTCAACGCCT |
| ***Hoxa11*** | TTCCGGCCACACTGAGGACAAG |
|  | ACTCTCGCTCCAGCTCTCGGATCT |
| ***Olig2*** | CGCAGCGAGCACCTCAAATCTAA |
|  | CCCAGGGATGATCTAAGCTCTCGAA |
| ***Ngn1*** | ATCACCACTCTCTGACCC |
|  | GAGGAAGAAAGTATTGATGTTGCCTTA |
| ***Ezh2*** | TTACTGCTGGCACCGTCTGATGTG |
|  | TGTCTGCTTCATCCTGAGAAATAATCTCC |
| ***Ezh1*** | AATATGGGAGCAAAGGCTCTGTATGTG |
|  | CACGAAGTTTCTTCCACTCTTCATTGAG |
| ***Eed*** | GCACAGAGATGAAGTTCTGAGTGCTG |
|  | ATAAGACTCCTTAATTGCATTCATCATCCT |
| ***18s*** | GTAACCCGTTGAACCCCATT |
|  | CCATCCAATCGGTAGTAGCG |
| ***Mash1*** | GCAACCGGGTCAAGTTGGT |
|  | GTCGTTGGAGTAGTTGGGGG |
| ***Id2*** | ATGAAAGCCTTCAGTCCGGTG |
|  | AGCAGACTCATCGGGTCGT |
| ***Dcx*** | CATTTTGACGAACGAGACAAAGC |
|  | TGGAAGTCCATTCATCCGTGA |
| ***Bmp4*** | CACTGTGAGGAGTTTCCATCACGAAG |
|  | GGATGCTGCTGAGGTTGAAGAGGA |
| ***Bmp6*** | GCCATCTCGGTTCTTTACTTCGAT |
|  | GTGGTTTAAGGCAGATGTTGTTGTT |
| ***Ngn2*** | TCGCCAGGGACTGTATCT |
|  | CTGTGAAGTGGAGTCCG |
| ***p21 (Cdkn1a)*** | CCTGGTGATGTCCGACCTG |
|  | CCATGAGCGCATCGCAATC |
| ***p27 (Cdkn1b)*** | TCAAACGTGAGAGTGTCTAACG |
|  | CCGGGCCGAAGAGATTTCTG |
| ***p57 (Cdkn1c)*** | CGAGGAGCAGGACGAGAATC |
|  | GAAGAAGTCGTTCGCATTGGC |
| ***Cdkn2a*** | CAGACCGACGGGCATAGCTTCA |
|  | GGATTTAGCTCTGCTCTTGGGATTGG |
| ***Bmp7*** | TTCCTGGTAACCGAATGCTGA |
|  | CCTGAATCTCGGCGACTTTTT |

**Supplemental Table 3. Primers for ChIP-qPCR**

| **Gene** | Sequence (5'-3') |
| --- | --- |
| ***Olig2*** | GCCTGACGCTACAGTGACAA |
|  | GGCTAATTCCGCTCAATGAA |
| ***Gata6*** | CGGTCCTTCGCTTTAGAAGATTGTAGG |
|  | ACACAGACCCAGGCAAGATAGCAAGA |
| ***Ngn1*** | CATTGTTGCGCGCCGTA |
|  | GCGATCAGATCAGCTCCT |
| ***Actin (promoter)*** | CCCAACACACCTAGCAAATTAGAACCAC |
|  | CCTGGATTGAATGGACAGAGAGTCACT |
| ***Actin (intron I)*** | CGTATTAGGTCCATCTTGAGAGTACACAGTATT |
|  | GCCATTGAGGCGTGATCGTAGC |
| ***Hoxa11*** | GCTGCGAAGAAGGTGCTGAACG |
|  | CGGTGGGTGAGGGATACTCTCTGG |
| ***Sox17*** | CCACTCACTCTGAGGCTCGCTGTAG |
|  | CCAAAGCAGACCTGAGGCTCGAA |

**Supplemental Table 4. Antibodies for western blot**

| Antigen | Dilution | Host | Manufacturer Cat. |
| --- | --- | --- | --- |
| p21 | 1:1000 | Rabbit | Abcam ab109199 |
| Eed | 1:5000 | Mouse | Millipore 05-1320 |
| Gata6 | 1:200 | Rabbit | Abcam ab22600 |
| β-actin | 1:5000 | Rabbit | Abcam ab8227 |

**Supplemental references**

Hwang WW, Salinas RD, Siu JJ, Kelley KW, Delgado RN, Paredes MF, Alvarez-Buylla A, Oldham MC, Lim DA. 2014. Distinct and separable roles for EZH2 in neurogenic astroglia. eLife 3:e02439.

Mori T, Tanaka K, Buffo A, Wurst W, Kuhn R, Gotz M. 2006. Inducible gene deletion in astroglia and radial glia--a valuable tool for functional and lineage analysis. Glia 54:21-34.

Su IH, Basavaraj A, Krutchinsky AN, Hobert O, Ullrich A, Chait BT, Tarakhovsky A. 2003. Ezh2 controls B cell development through histone H3 methylation and Igh rearrangement. Nat Immunol 4:124-131.
