# Supplementary figures and images for: Polycomb Protein Eed is Required for Neurogenesis and Cortical Injury Activation in the Subventricular Zone
Source: Cereb Cortex. 2018 Feb 3;28(4):1369–82. doi: 10.1093/cercor/bhx289 (PMC6093351; doi:10.1093/cercor/bhx289)

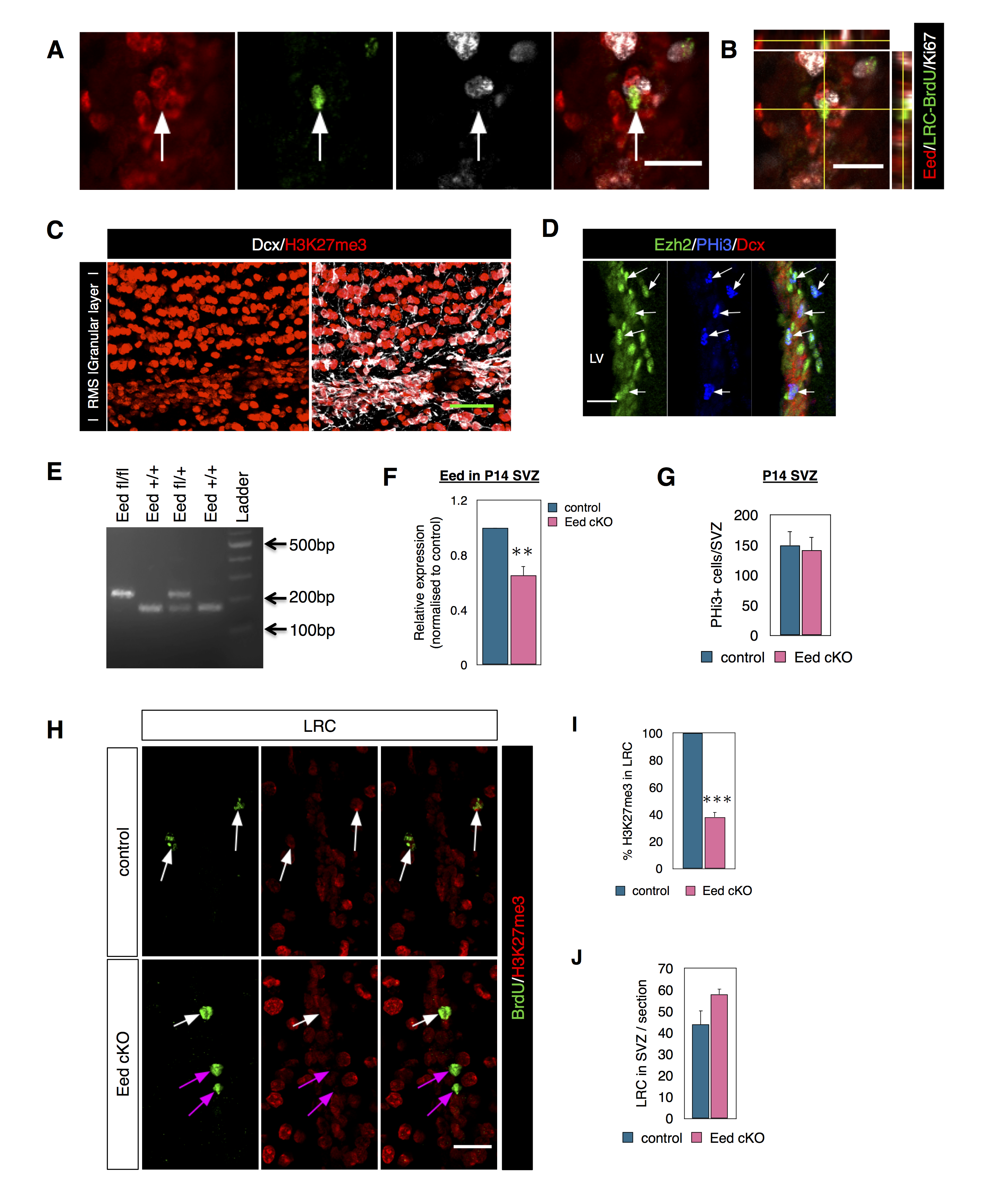

Supplement: Supplementary Data [file bhx289_supplementary-materials.zip › FIGURES1.tiff]

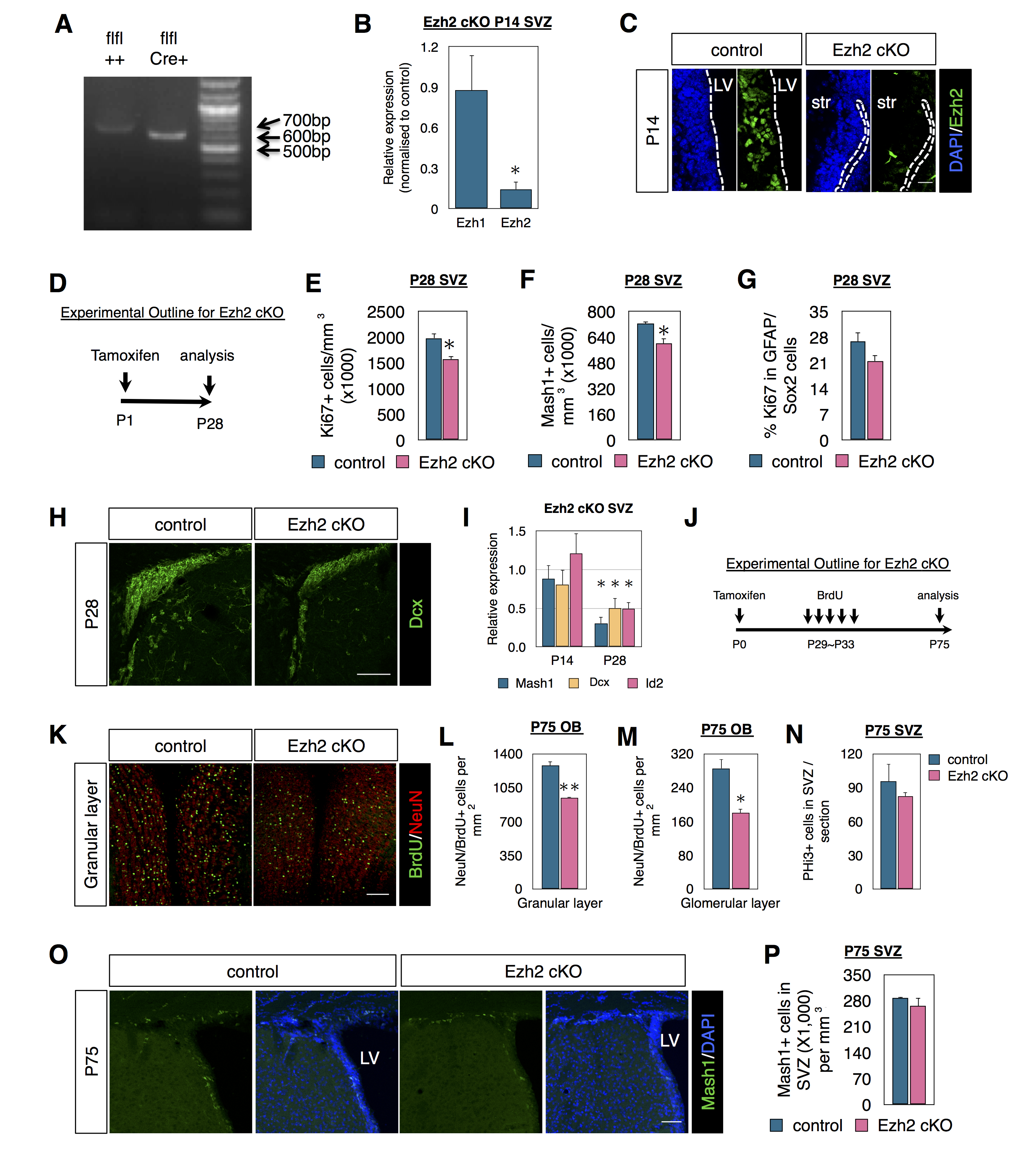

Supplement: Supplementary Data [file bhx289_supplementary-materials.zip › FIGURES2.tiff]

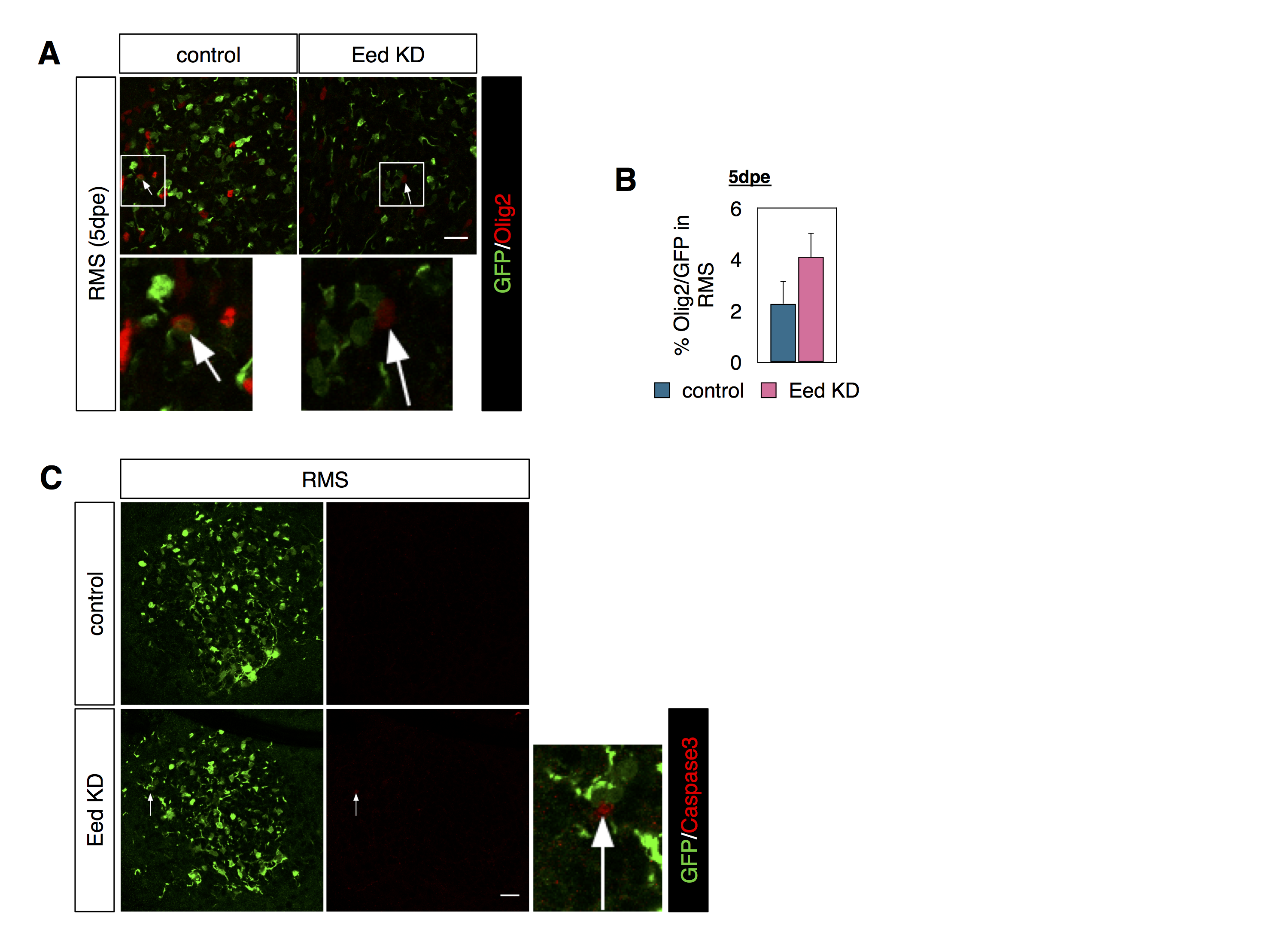

Supplement: Supplementary Data [file bhx289_supplementary-materials.zip › FIGURES3.tiff]

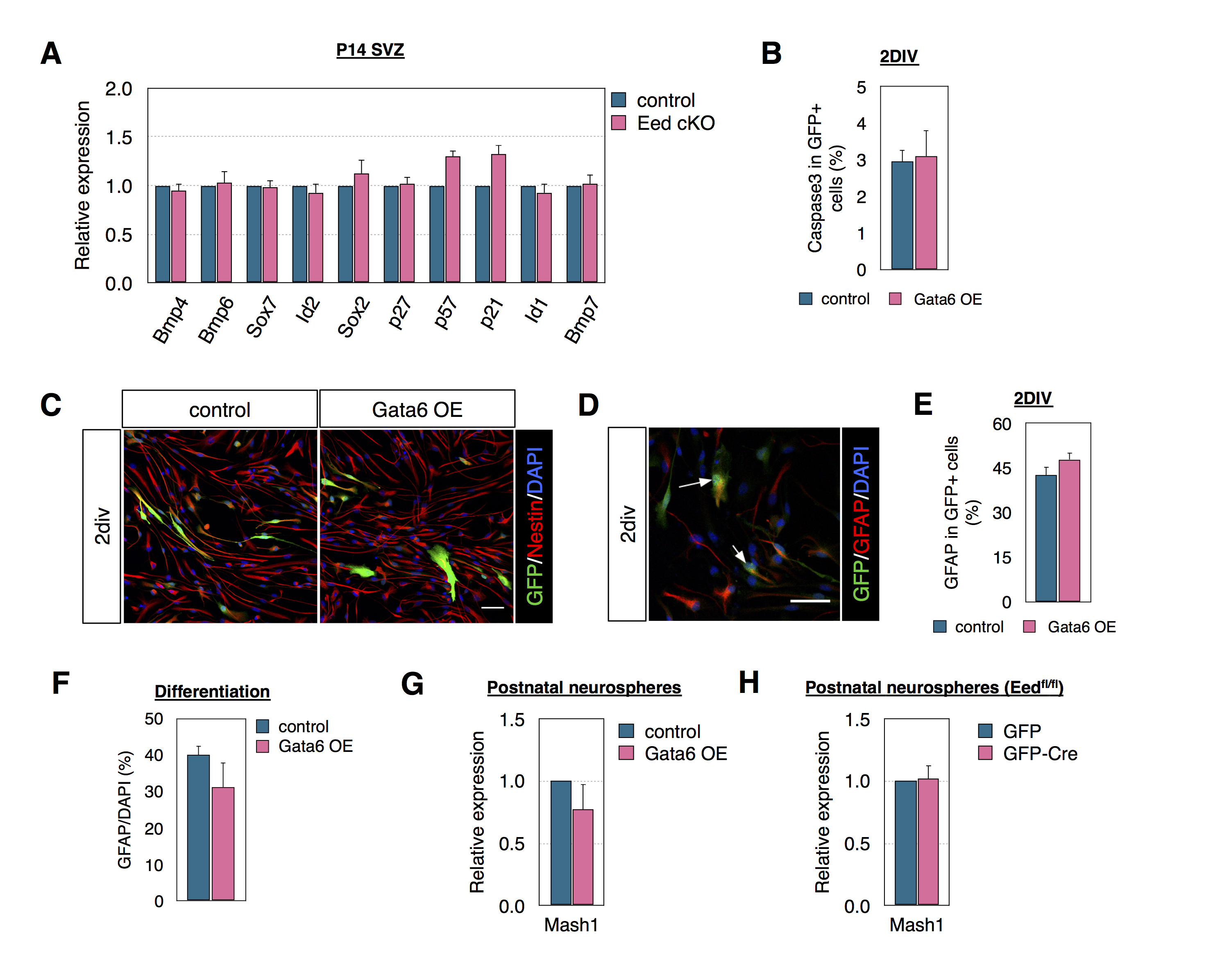

Supplement: Supplementary Data [file bhx289_supplementary-materials.zip › FIGURES4.tiff]

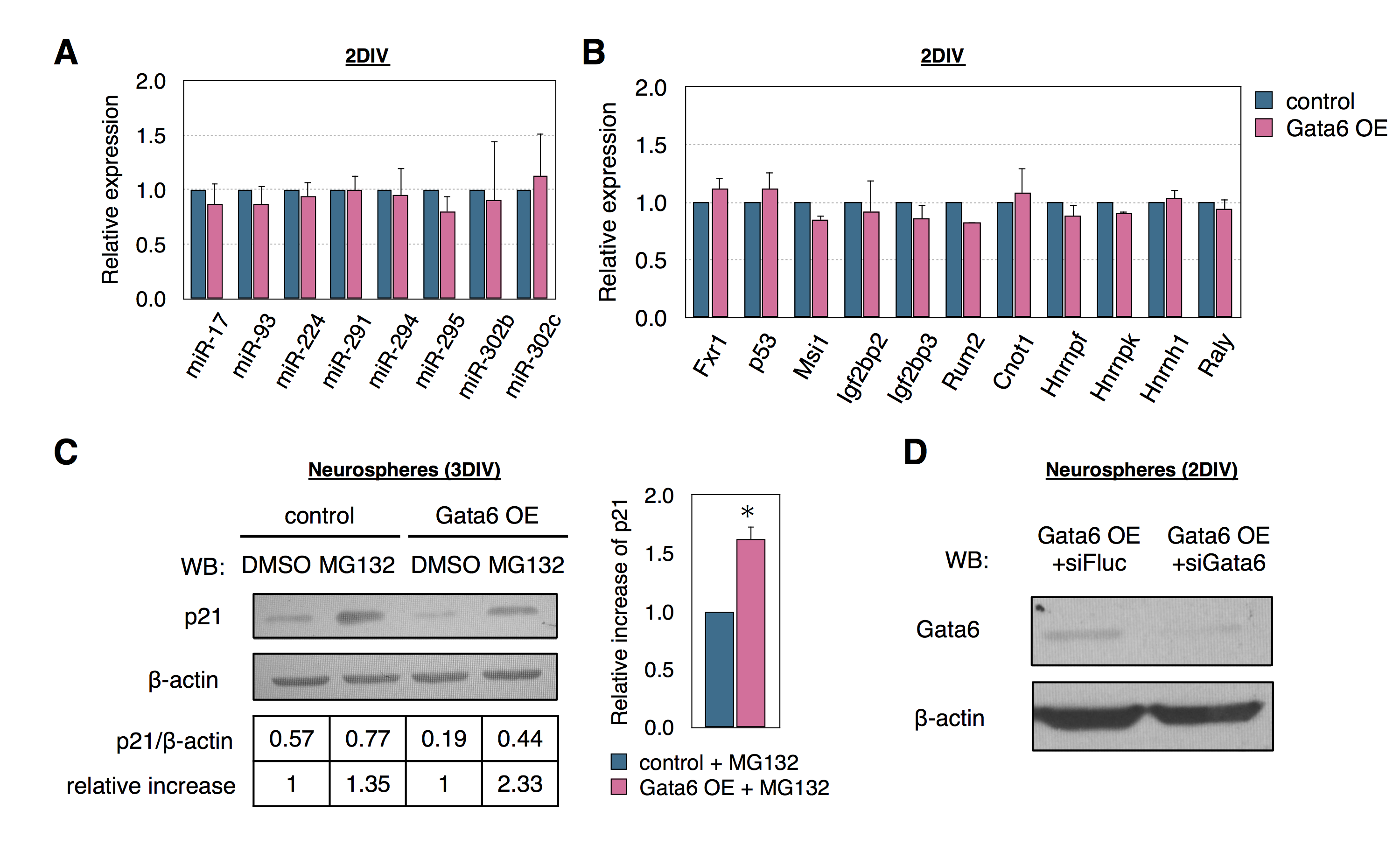

Supplement: Supplementary Data [file bhx289_supplementary-materials.zip › FIGURES5.tiff]
